# The membrane-to-cortex distance regulates mDia1 activity to control cortical mechanics

**DOI:** 10.1038/s41467-026-72845-3

**Published:** 2026-09-04

**Authors:** Léanne Strauss, Sergio Lembo, Samuel F. Gérard, Marc Siggel, Dorothy Cheng, Martin Bergert, Sarah K. Foster, Joseph Vermeil, Mauricio Toro-Nahuelpan, Lena M. Fischer, Qin Yu, Ewa Sitarska, Chii Jou Chan, Jan Kosinski, Matthieu Piel, Olivia du Roure, Julien Heuvingh, Julia Mahamid, Alba Diz-Muñoz

**Affiliations:** 1 Cell Biology and Biophysics Unit, European Molecular Biology Laboratory (EMBL), Heidelberg, Germany; 2 Molecular Systems Biology Unit, EMBL, Heidelberg, Germany; 3 EMBL Hamburg, Hamburg, Germany; 4 Centre for Structural Systems Biology (CSSB), Hamburg, Germany; 5 Institut Curie, PSL University, CNRS, Paris, France; 6 Institut Pierre Gilles de Gennes, PSL University, CNRS, Paris, France; 7 Physique et Mécanique des Milieux Hétérogènes (PMMH), CNRS, ESPCI Paris–Université PSL, Sorbonne Université, Université Paris Cité, Paris, France; 8 Developmental Biology Unit, EMBL, Heidelberg, Germany; 9 Present Address: Institute of Science and Technology Austria (ISTA), Klosterneuburg, Austria; 10 Present Address: Laboratoire Jean Perrin, Institut de Biologie Paris-Seine, Sorbonne Université, 11 CNRS UMR, Paris, France; 11 Present Address: Institut Jacques Monod, CNRS UMR, Paris, France; 12 Present Address: Computational Innovation, Boehringer Ingelheim Pharma GmbH & Co. KG, Biberach, Germany; 13 Present Address: The Francis Crick Institute, London, UK; 14 Present Address: Department of Cell Biology, Harvard Medical School, Boston, MA USA; 15 Present Address: Program in Cellular and Molecular Medicine, Boston Children’s Hospital, Boston, MA USA; 16 Present Address: Mechanobiology Institute, Department of Biological Sciences, National University of Singapore, Singapore, Singapore

**Keywords:** Biophysics, Actin

## Abstract

The shape of animal cells is controlled by their surface, which comprises the cell cortex, a peripheral actin network, tethered to the plasma membrane by membrane-to-cortex attachment proteins. Changes in cortical components have long been considered to dominate the regulation of forces and mechanical properties at the cell surface and drive morphogenesis. Here, we show that the coupling of the cortex to the membrane is also key for the regulation of its mechanical properties. By combining molecular engineering with biophysical approaches and in-cell cryo-electron tomography we describe the cell surface with nanometer-resolution and link its organization to cell-scale mechanics. We find that membrane-to-cortex attachment proteins can physically draw the cortex closer to the membrane, in a density and length-dependent manner. This reduction of the membrane-to-cortex distance controls the activity of the formin mDia1, leading to a reduction in cortical tension. Our study thus defines a novel mechanism whereby the membrane-to-cortex distance is a functional geometrical parameter that regulates cell surface properties.

## Abstract (web summary)

The surface of mammalian cells is a multi-layered composite structure. The authors combine advanced biophysical methods with cryo-electron tomography to identify the gap between the membrane and the actin cortex as a critical geometrical parameter that controls cell surface mechanics.

## Introduction

The animal cell surface comprises the plasma membrane linked to the underlying actin cortex by specialized membrane-to-cortex attachment proteins^1^. Together these components form a composite interface, with emergent mechanics that could not be predicted by the simple combination of their individual properties^2,3^. This composite nature is critical for ensuring that the structure and integrity of the cell surface are maintained while simultaneous dynamic processes take place. This functional plasticity is particularly remarkable during complex processes that require cell shape change, such as mitosis and cell migration. Deciphering the control of cell surface mechanics is thus essential to understand morphogenesis.

Changes in the localisation, architecture, and activity of cortical components are often considered the main drivers of cell surface mechanics. The cortex comprises a thin network of actin filaments, myosin motors, and actin-binding proteins. Its structure and the subcellular distribution of its components vary substantially across different cell types and physiological conditions^4^. Active forces generated by myosin motors are key to the contractile stresses present in the cortex. Additionally, numerous other factors influence cortical mechanics, including actin turnover and the cortical architecture. These are controlled by a large and diverse group of actin nucleating, branching, crosslinking, and severing proteins^5–8^. Cortical actin filaments are nucleated by both the Actin Related Protein 2/3 (Arp2/3) complex and the Diaphanous-related Formin-1 (mDia1)^6^. The resulting respective branched or linear filaments have different lengths, effective turnover rates, and sensitivity to myosin activity^9,10^. Hence, the proportion and spatial distribution of these nucleators play a key role in determining the mechanical properties of the resulting cortical network^9,11^.

However, how cortical organization is regulated during key cellular processes remains poorly understood. While cortical mechanics have been dissected using a plethora of methods (reviewed in ref. ^12^), analysing the 3D architecture of the actin network in cells is a long-standing challenge owing to the poor preservation of actin filaments during chemical fixation in traditional room temperature microscopy methods^13,14^. Nevertheless, the combination of light and electron microscopy approaches have shed some light on the architecture of the actin cytoskeleton at the basal surface and migration front in some cell types^15,16^. Moreover, the cell cortex has been visualized in cells and cellular blebs using scanning electron microscopy (SEM) of detergent-treated samples^5,17,18^, and with cryogenic electron microscopy (cryo-EM) in cellular blebs^19^. However, to our knowledge, there is no data that describes the intact cortical network in 3D.

Furthermore, the cortex does not function in isolation; it is mechanically coupled to the plasma membrane by membrane-to-cortex attachment proteins, the best studied family of which is the ERM family (ezrin, radixin, and moesin). ERM proteins are key regulators of membrane mechanics, in particular membrane tension^1^, and play important signalling roles in a plethora of processes^20^. They are activated by phosphorylation, which induces a conformational change that exposes the actin-binding domain^21–23^. ERM proteins distribute asymmetrically during morphogenetic processes, coinciding with mechanically and functionally distinct cortical regions in cells: they specifically localise to the rear of migrating cells^24^, the cytokinetic ring of dividing cells^25,26^, the microvillar domain of mouse oocytes^27^, and the apical domain of early mouse blastomeres^28^ and epithelial cells^29^. ERM proteins have been shown to play a key role in the establishment and maintenance of these specialized cortical domains but whether and how changes in ERM protein density affect the actin cortex and its mechanics remains unclear.

In this study, we show how the physical coupling of membrane and cortex is crucial for the regulation of actin nucleation and therefore for the control of cortical mechanics. Specifically, we identify the membrane-to-cortex distance as a key tuneable parameter that can gate the activity of mDia1. We employ cellular cryo-electron tomography (cryo-ET) to image the cortex with single-filament resolution, and molecular engineering together with biophysical approaches to elucidate the mechanical and structural consequences of manipulating membrane-to-cortex attachment. We show that a high density of membrane-to-cortex attachment proteins can narrow the gap between membrane and cortex, in a manner that also depends on their length. This constriction physically restricts the activity of the formin mDia1, resulting in a reduction in cortical tension. We propose that this purely geometrical mechanism is key for patterning cell mechanics via the local control of actin nucleation.

## Results

### Membrane-to-cortex attachment proteins regulate cortical tension

**Fig. 1:**
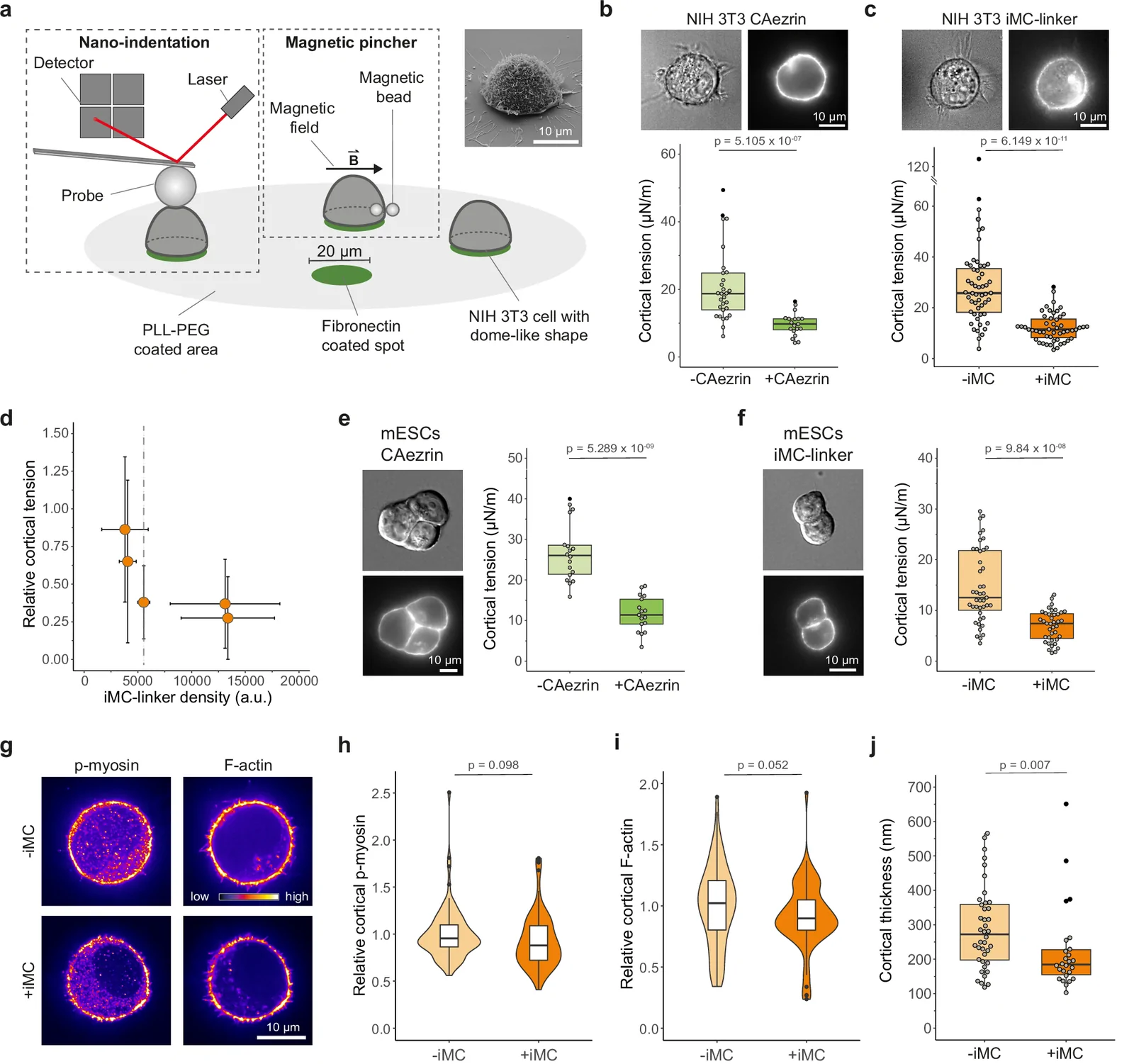
Membrane-to-cortex attachment regulates cortical tension and thickness. **a** Experimental setup for cortical tension and thickness measurements in micropatterned NIH 3T3 fibroblasts by nano-indentation and magnetic pincher; B = magnetic field vector. Insert: representative SEM image showing the XY view with a 54° tilt angle of a micropatterned cell. **b**, **c** Representative bright-field and epifluorescence images of NIH 3T3 fibroblasts (top) expressing mCherry-tagged CAezrin (**b**) or the iMC-linker (**c**); cortical tension upon expression of the respective construct (bottom); n_Cells_: -CAezrin = 29, +CAezrin = 19, -iMC = 52, +iMC = 51; n_Exp_: CAezrin = 3, iMC = 5. **d** Cortical tension of various iMC-linker-expressing clonal lines normalized to the respective non-induced control. Dots represent the median of independent experiments of individual clonal line (n_Exp_≥2, error bars show the standard error of the mean; n_Cells_ > 18 for individual clonal line per condition; for exact numbers see Supplementary Table 1. Dashed line indicates the clonal cell line showing a reduction in cortical tension by more than 50%. All clonal cell lines used in this study for further experiments express the respective construct at similar or higher levels, a.u. = arbitrary units. **e**, **f** Representative differential interference contrast and epifluorescence images of mESCs (left) expressing CAezrin (**e**) or iMC-linker (**f**); cortical tension upon expression of the respective construct (right); n_Cells_: -CAezrin = 18, +CAezrin = 17, -iMC=43, +iMC = 39; n_Exp_: CAezrin = 3, iMC = 4. **g** Representative confocal XY plane of fixed ±iMC NIH 3T3 fibroblasts stained for p-myosin and F-actin. **h**, **i** Mean fluorescence intensity of cortical p-myosin (**h**) and F-actin (**i**), normalized per replicate to mean fluorescence intensity of -iMC cells; n_Cells_: -iMC = 89, +iMC = 74; n_Exp_ = 3. The coloured area in the violin plots reflects the density distribution of data points. For details of embedded box plot see below. **j** Cortical thickness in ±iMC NIH 3T3 fibroblasts; n_Cells_: -iMC = 40, +iMC = 26; n_Exp_ = 4. Box plots show the median (centre horizontal line), the lower and upper hinges correspond to the first and third quartiles (the 25th and 75th percentiles), whiskers extend from the hinge to the largest/smallest value, but no further than 1.5x interquartile range, outliers are marked by a black dot, dots represent the mean of multiple measurements per single cell. n_Cells_= number of analysed cells of each condition, n_Exp_= number of independent experiments. Normality of data distribution was tested by Shapiro-Wilk test. Two-tailed t-test was used for normally distributed data. Otherwise, a two-sided non-parametric Wilcox test was used. See also Supplementary Figs. 1 and 2, and Supplementary Table 1.

ERM proteins are known to be enriched at functionally distinct regions of the cell cortex with differential mechanical properties^24–29^. Thus, we first examined whether modulation of membrane-to-cortex attachment has a direct effect on the mechanical properties of the cortex. We increased membrane-to-cortex attachment by expressing an mCherry-tagged, constitutively-active version of ezrin (CAezrin, T567D^23^) in a doxycycline-inducible manner in NIH 3T3 fibroblasts (Fig. 1b top, Supplementary Fig. 1a, b). To keep a constant cell geometry between our measurements and induce the formation of a uniform cortical network^4^, we plated these cells on circular micropatterns, such that they acquire a dome-like shape (Fig. 1a). Cortical tension, a key mechanical property of the cortex, was measured by atomic force nano-indentation (Fig. 1a, Supplementary Fig. 1c). Surprisingly, increasing membrane-to-cortex attachment by CAezrin expression led to a decrease in cortical tension (Fig. 1b bottom).

In order to understand the origin of this phenotype, we first needed to disentangle whether it arose from the mechanical or signalling roles of ERM proteins. Beyond tethering the plasma membrane to the cortex, ERM proteins can act as signalling intermediates in diverse biochemical pathways independently of their role in cell mechanics (reviewed in ref. ^20^). As the expression of CAezrin is likely to exhibit additional effects unrelated to membrane-to-cortex attachment, we next used our inert membrane-to-cortex linker (iMC-linker), a synthetic molecular tool that mimics the domain architecture of endogenous membrane-to-cortex attachment proteins but is inert with regard to signalling^30^. Specifically, the iMC-linker is reduced to three small protein domains: the CH1/CH2 domain of utrophin^31^ to bind filamentous actin (F-actin), the lipidation motif of lyn^32^ to insert into the plasma membrane, and the fluorescent protein mCherry to link the two domains and visualize the linkers’ subcellular localisation (Fig. 1c top, Supplementary Fig. 1d). Consistent with the effect of CAezrin, increasing membrane-to-cortex attachment by expression of the iMC-linker (Supplementary Fig. 1e) also significantly decreased cortical tension in fibroblasts (Fig. 1c bottom). By measuring several cellular clones with varied levels of iMC-linker expression, we found that this reduction followed a density-dependent trend (Fig. 1d). Thus, our results show that membrane-to-cortex attachment not only contributes to the mechanical properties of the membrane as previously reported^1^ (see also Supplementary Fig. 1b, e), but also to those of the actomyosin cortex.

Expression of the iMC-linker did not change the phosphorylation level of endogenous ERM proteins, suggesting that the observed effects do not result from changes in signalling associated with ERM activation or inactivation (Supplementary Fig. 2a, b). To rule out a change in actin dynamics by the actin-binding function of the iMC-linker, we expressed the actin-binding domain alone tagged with mCherry and found that it was not sufficient to decrease cortical tension (Supplementary Fig. 1f). Similarly, the expression of mCherry with a lipidation motif of lyn also failed to decrease cortical tension (Supplementary Fig. 1g), altogether demonstrating that the tethering of cortical actin to the membrane is key for this mechanical phenotype.

We next tested the effect of iMC-linker expression in cells in suspension, to check that this effect was not dependent on cell adhesion^33^. Regardless of cell adhesion to the substrate, iMC-linker-expressing cells had lower cortical tension (Supplementary Fig. 1h). Finally, we examined this effect in a different cell model by overexpressing CAezrin or the iMC-linker in naïve mouse embryonic stem cells (mESCs) (Fig. 1e, f left, Supplementary Fig. 1i, j). Nano-indentation measurements consistently showed a decrease in cortical tension (Fig. 1e, f right, Supplementary Fig. 1k). This demonstrates that the regulation of cortical tension by membrane-to-cortex attachment is a consistent feature across different cell types.

Cortical tension has been shown to depend on actin architecture^5^ and the activity of myosin-2 motors which, upon phosphorylation, pull on the actin filaments to contract the network^34^. The two parameters are also interrelated, as aligned filament architectures make it easier for myosin-2 to contract the cortical network^35^. Thus, the observed decrease in tension upon increased membrane-to-cortex attachment could result from reduced myosin-2 activity and/or changes in cortical actin architecture. To explore these two possibilities, we first quantified active myosin-2 at the cell cortex in iMC-linker-expressing cells. Analysis of a myosin-2 reporter^36^ in live cells showed a minor reduction in cortical myosin levels in the +iMC condition (Supplementary Fig. 2c, d), while staining for phosphorylated myosin-2 (p-myosin) in fixed cells showed no significant effects (Fig. 1g, h). Overall, we concluded that this small effect alone was unlikely to explain the two-fold reduction in cortical tension. We next quantified the amount of F-actin at the cortex in fixed and live cells, and found a non-significant decrease in cortical F-actin in +iMC cells as compared to control cells (Fig. 1g, i, Supplementary Fig. 2e, f). Finally, using a magnetic pincher^37^ we observed a 30% reduction in cortical thickness, with no concomitant change in the elasticity of the cortical layer per se (Fig. 1a, j, Supplementary Fig. 1l).

Taken together, our results suggest that increasing membrane-to-cortex attachment reduces cortical tension by changing the cortical network in a non-intuitive way. This highlights the complexity of the cell surface composite interface and the necessity of a precise structural understanding of the different components. Therefore, we next examined whether increased membrane-to-cortex attachment linker protein expression leads to changes in cortical organization.

### Membrane-to-cortex attachment proteins reduce the membrane-to-cortex distance in a length-dependent manner

Determining the architecture of actin networks in cells is a long-standing challenge^13,14^. Nevertheless, insights into basal actin structures and cellular blebs have been gained by combining light and (cryogenic) electron microscopy approaches^15–17,19,38^. However, the three-dimensional organization of the cell cortex remains unknown, a challenge compounded by its diffraction-limited thickness^5,39^, high density of actin filaments^18^, and close proximity to the membrane.

**Fig. 2:**
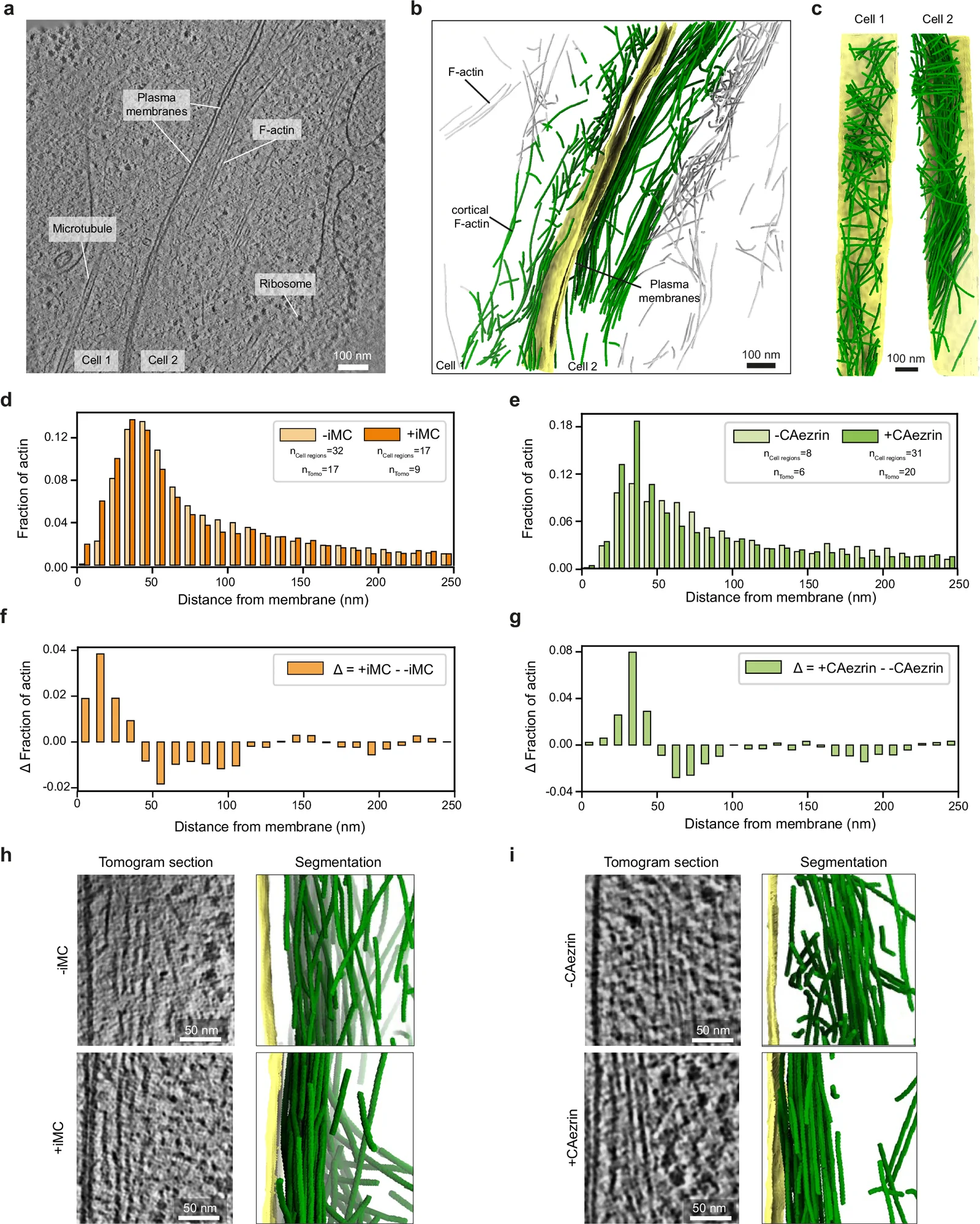
Membrane-to-cortex attachment regulates the spatial distribution of cortical actin filaments. **a**, **b** Representative tomographic slice through two adjacent NIH 3T3 fibroblast cellular regions (Cell 1, Cell 2) (**a**) and corresponding segmentation of actin filaments (green and grey) and plasma membrane (yellow) (**b**). See Supplementary Video 1 for complete tomogram. Actin filaments within 250 nm of the membrane (green) were taken as cortical actin. **c** Orthogonal views of tomogram shown in (**b**), showing segmented cortical actin filaments within 50 nm of the plasma membrane. **d**, **e** Spatial distribution of cortical actin upon expression of the iMC-linker (**d**) or CAezrin (**e**). Displayed is the fraction of actin as a function of distance from the plasma membrane, bars depict average actin fraction across all cellular regions of one condition. n_Cells_: -iMC = 15, +iMC = 9, -CAezrin = 7, +CAezrin = 15. **f**, **g** Difference (Δ) in actin fraction based on data shown in d and e, calculated between cells expressing the construct and their respective control cells. Data are displayed as a function of distance from the plasma membrane. **h**, **i** Close-up of the membrane-to-cortex interface in representative tomographic slices (left), and the corresponding segmentations of actin filaments (green) and plasma membrane (yellow) (right), for the iMC-linker (**h**) and CAezrin (**i**) expressing cells. n_Cell regions_= number of analysed cellular regions (1-2 in each tomogram), n_Tomo_= number of tomograms, we obtained 1-4 tomograms per lamella, due to the high variability between tomograms (Supplementary Figs. 5 and 6), cellular regions in tomograms from a single lamella were considered independent data points, n_Cells_= number of analysed cells. See also Supplementary Table 3, Supplementary Figs. 3–6 and Supplementary Video 1.

Thus, to assess how membrane-to-cortex attachment proteins affect the actin cortex, we established a cryo-ET workflow to resolve actin filaments at the cell surface in 3D and at the nanometer-scale. NIH 3T3 fibroblasts were seeded on micropatterned electron microscopy (EM-) grids, and displayed the same reduction in cortical tension as in our measurements of cell surface mechanics described above (Supplementary Fig. 3a). To visualize the cortex at the rounded parts of the cell, we used cryo-focused ion beam milling (cryo-FIB)^40^ to produce ~200 nm thin lamellae in the equatorial plane of the plunge-frozen cells. To ensure structural preservation of the cell surface in the lamellae, the micropatterns were designed to allow the plating of two cells in proximity to each other (Supplementary Fig. 3b, c). The cells did not form cell-cell adhesions during growth, but came into contact only during the freezing step. Lamellae were generated across two cells growing on adjacent micropatterns, such that each tomogram contained two membranes and two cortices^41^ (Fig. 2a, Supplementary Fig. 3d–f). We generated 3D reconstructions at 1.3 nm/pixel and semi-automatically segmented individual actin filaments and the plasma membranes (Fig. 2a–c, Supplementary Fig. 3f, g, Supplementary Video 1).

As a comprehensive structural description of the actin cortex in interphase cells has not been previously reported, we first set out to examine the general characteristics of the cortex, which exhibited variability between tomograms (Supplementary Fig. 4). While visual inspection of our tomograms revealed a clear peak in actin density proximal to the membrane, defining where the cortex ends was challenging. Moreover, cells also contain internal actin structures, and there are no clear structural or compositional criteria to distinguish them from the cortex^4^. Thus, based on the filament density plot (Supplementary Fig. 3h) and the size of our tomograms, we considered actin filaments within 250 nm of the membrane to be part of the cell cortex. Filaments in the cortical space were frequently aligned parallel to the plasma membrane (Fig. 2b, Supplementary Fig. 4), and branch points and bundles were visible (Supplementary Fig. 5a, b). However, in striking contrast to previously described actin networks in lamellipodia^42^, actin branching was overall rare in the analysed cortices (mean = 0.1/µm F-actin; Supplementary Fig. 5c). Additionally, there were significant areas of bare membrane (Fig. 2c, Supplementary Fig. 4 and Supplementary Fig. 6).

Next, we compared cortical parameters among control, CAezrin-expressing and iMC-linker-expressing cells. The number of branches and the bundling parameter were not quantifiably different in the +iMC or +CAezrin conditions as compared to controls (Supplementary Fig. 5c, d). However, our analysis showed that expression of CAezrin or the iMC-linker changed the distribution of actin filaments in the cortex (Fig. 2d–g, Supplementary Fig. 5e, f). In particular, both conditions increased the probability of finding actin within 50 nm of the membrane compared to control cells, while decreasing the probability beyond 50 nm (Fig. 2f, g).

**Fig. 3:**
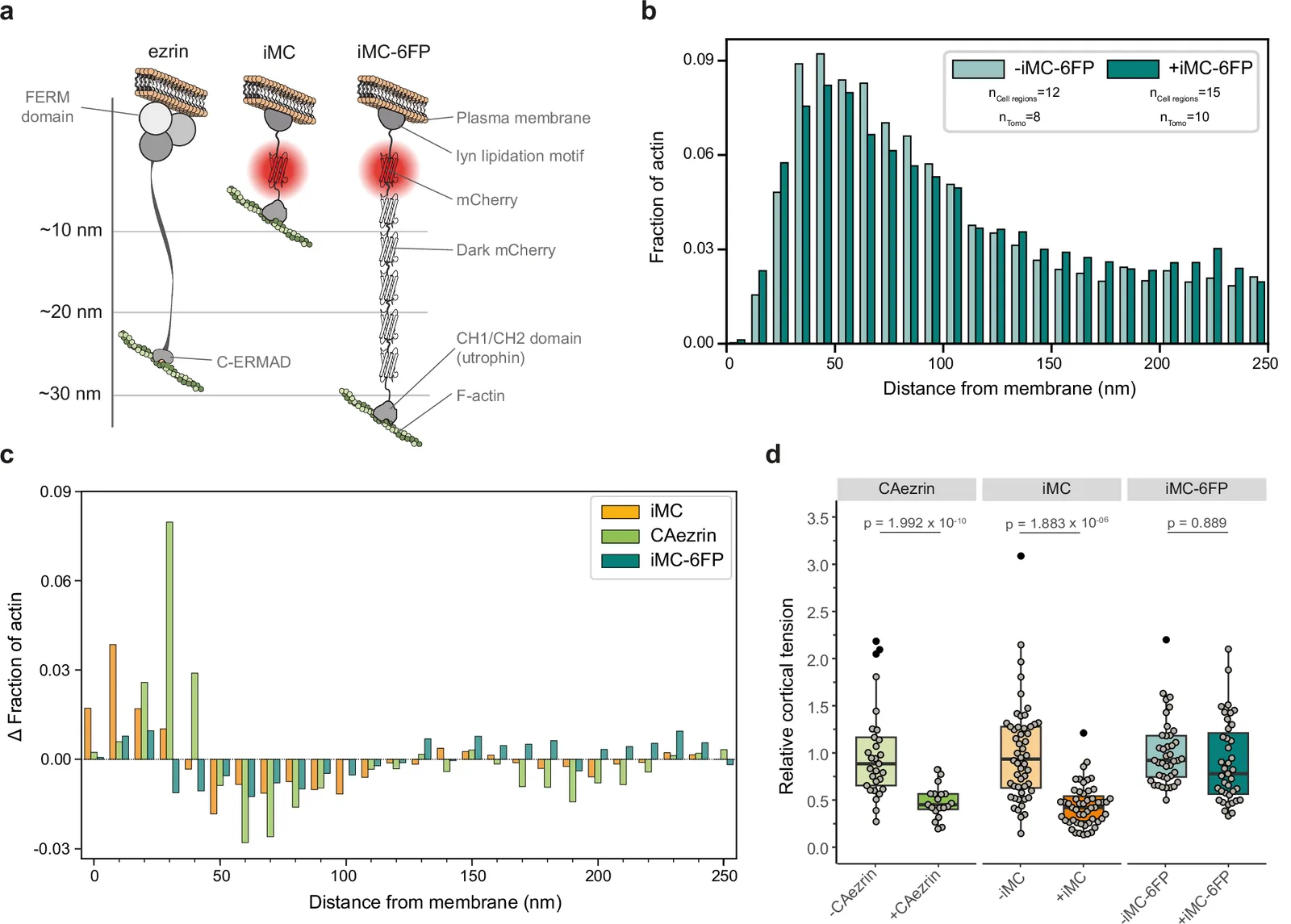
Membrane-to-cortex attachment protein length and density control the membrane-to-cortex gap. **a** Schematic representation of the estimated length upon full extension of CAezrin^72^, the iMC-linker, and the iMC-6FP-linker. **b** Spatial distribution of cortical actin upon expression of the iMC-6FP-linker in NIH 3T3 fibroblasts analysed by cryo-ET. Displayed is the fraction of actin as a function of distance from the plasma membrane. Bars depict average fraction of actin across all cellular regions of one condition. n_Cells_: -iMC−6FP = 6, +iMC-6FP = 10. **c** Difference (Δ) of actin fraction for the iMC-linker, CAezrin and the iMC-6FP-linker (Δ for the iMC-linker and CAezrin from Fig. 2f, g, respectively, shown here for better comparison). Δ for the iMC-6FP-linker is calculated between cells expressing the construct and respective control, based on data shown in b. Data are displayed as a function of distance from the plasma membrane. **d** Relative cortical tension in NIH 3T3 fibroblasts expressing CAezrin, the iMC-linker (normalized data from Fig. 1b, c, respectively) or the iMC-6FP-linker, normalized per replicate to the mean of the respective control; n_Cell_: -iMC-6FP = 40, +iMC-6FP = 39; n_Exp_: iMC-6FP = 4. Box plots show the median (centre horizontal line), the lower and upper hinges correspond to the first and third quartiles (the 25th and 75th percentiles), whiskers extend from the hinge to the largest/smallest value, but no further than 1.5x interquartile range, outliers are marked by black dots, dots represent the mean of multiple measurements per single cell. n_Cell regions_= number of analysed cellular regions (1–2 in each tomogram), n_Tomo_= number of tomograms, we obtained 1-4 tomograms per lamella, due to the high variability between tomograms, cellular regions in tomograms from a single lamella were considered independent data points (Supplementary Figs. 4 and 6), n_Cells_= number of analysed cells, n_Exp_= number of independent experiments. Normality of data distribution was tested by Shapiro-Wilk test. Statistical significance was calculated using a two-sided non-parametric Wilcox test. See also Supplementary Fig. 7 and Supplementary Fig. 2a, b.

These findings suggest that expression of CAezrin or the iMC-linker decreased the distance between the membrane and the most peripheral actin filaments (Fig. 2f-i, Supplementary Fig. 5e, f). In fact, the shorter iMC-linker seemed to pull the actin filaments even closer to the membrane, shrinking this gap more than CAezrin (Fig. 3a and 2f, g). As nanometre-scale protein geometry has been shown to regulate diverse cellular functions^43–45^, we aimed to discern whether the reduction in cortical tension was caused by the increase in membrane-to-cortex attachment or the reduction in the membrane-to-cortex distance. To that end, we generated an iMC-like-linker with six mCherry moieties (one fluorescent and five dark mCherrys) between the membrane-binding and actin-binding domains (henceforth referred to as the iMC-6FP-linker). This protein is estimated to span more than 30 nm when fully extended, slightly longer than the predicted length of ezrin (Fig. 3a). While tether pulling experiments confirmed that cells expressing the iMC-6FP-linker had increased membrane-to-cortex attachment comparable to cells expressing similar levels of the iMC-linker or CAezrin (Supplementary Fig. 1b, e, Supplementary Fig. 7a), tomograms of +iMC-6FP cells showed an actin filament distribution similar to that of control cells (Fig. 3b, c, Supplementary Fig. 7b–d). Thus, the expression of a longer linker no longer closed the gap to the membrane, but it also did not push the actin cortex away. Importantly, +iMC-6FP cells showed no change in cortical tension when compared to control cells (Fig. 3d), suggesting that the reduction of the membrane-to-cortex gap is crucial for the observed change in cortical tension in +CAezrin and +iMC cells. Finally, we designed another linker construct, with four fluorescent proteins, making it slightly shorter in predicted length than full-length ezrin (henceforth referred to as the iMC-4FP-linker, Supplementary Fig. 7e). As expected, expression of the iMC-4FP-linker not only increased membrane-to-cortex attachment (Supplementary Fig. 7f), but also significantly reduced cortical tension (Supplementary Fig. 7g).

We conclude that when linkers are shorter than a certain threshold (25 nm in round interphase fibroblasts), a high density of linker proteins reduces the size of the membrane-to-cortex gap, which correlates with a reduction in cortical tension. As demonstrated by our experiments with the iMC-6FP-linker, high density of a longer linker alone is insufficient to cause either the change in cortical actin distribution or the reduction in cortical tension. Together, these results suggest that the distance between the membrane and the cortex regulates processes occurring at the plasma membrane that are key to understanding the mechanical phenotype observed in the +CAezrin and +iMC cells.

### Membrane-to-cortex attachment proteins dictate mDia1 activity via regulation of the membrane-to-cortex distance

Cortical actin filament nucleation happens at the membrane-to-cortex interface^46–48^. We therefore hypothesized that this process could be sensitive to the size of the membrane-to-cortex gap. Cortical actin filaments can be polymerized by formins de novo, or by the Arp2/3 complex from preexisting filaments^49^. In vitro, these nucleators generate distinct network topologies with different mechanical properties^50–52^. In cells, the cortex appears soft if predominantly nucleated by the Arp2/3 complex, and stiff if predominantly generated by formins. Importantly, formins have been shown to be primarily responsible for nucleating the long, tension-bearing filaments in the cortex^9^. The activity ratio of the two nucleators can thus control the network architecture and thereby the mechanics of the cell cortex^9,53^.

**Fig. 4:**
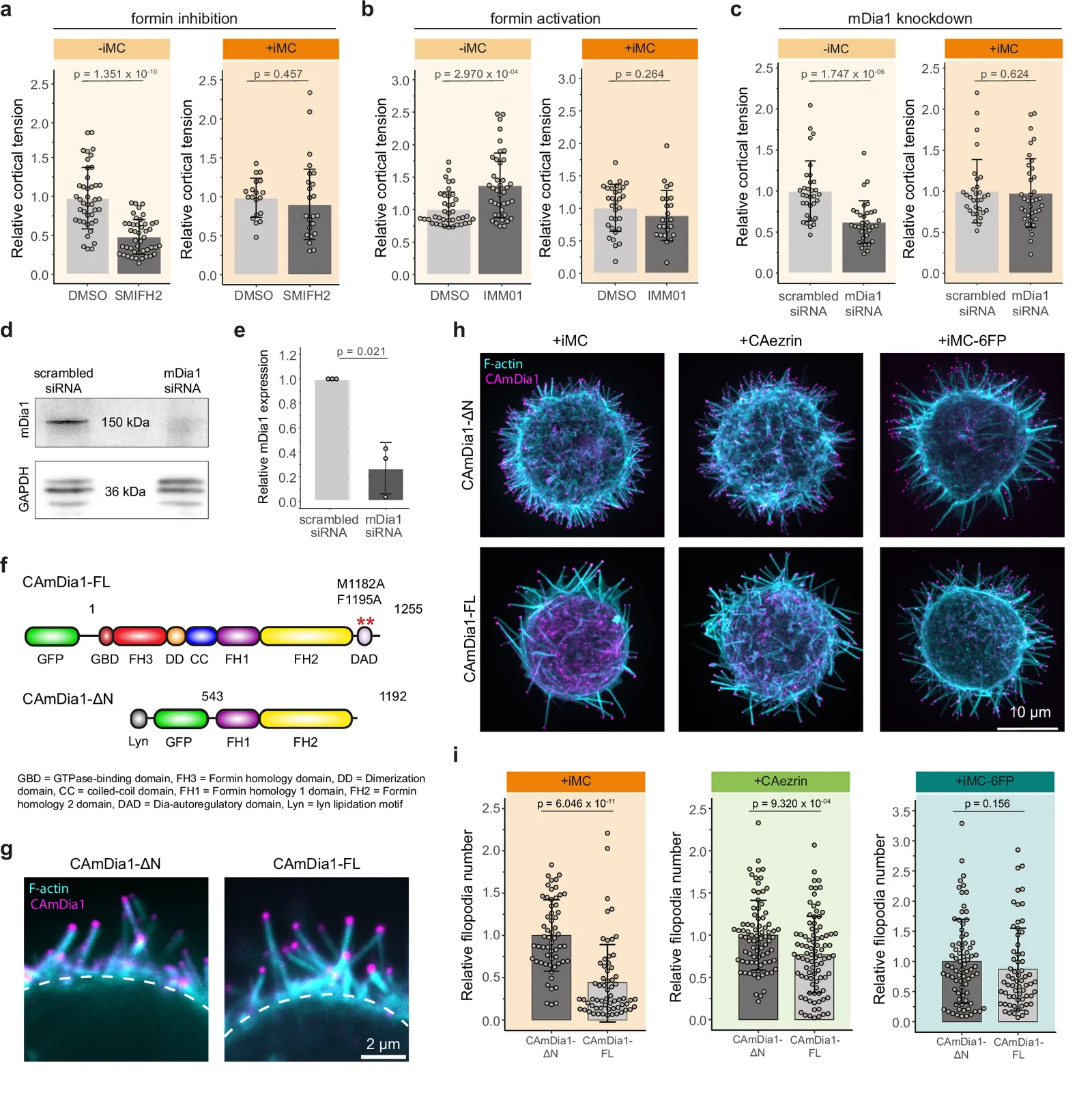
Reducing membrane-to-cortex distance interferes with mDia1 activity. **a**, **b** Relative cortical tension of ±iMC NIH 3T3 fibroblasts upon treatment with the pan-formin inhibitor SMIFH2^56^ (**a**) or the formin activator IMM01 (**b**), values are normalized per replicate to the mean cortical tension of respective vehicle only (DMSO) treated cells; n_Cells_(SMIFH2): -iMC+DMSO = 42, -iMC+SMIFH2 = 45, +iMC+DMSO = 19, +iMC+SMIFH2 = 22, n_Cells_(IMM01): -iMC+DMSO = 39, -iMC+IMM01 = 39, +iMC+DMSO = 31, +iMC+IMM01 = 24; n_Exp_(SMIFH2): -iMC=4, +iMC=2; n_Exp_ (IMM01): -iMC=4, +iMC=3. **c** Relative cortical tension of ±iMC NIH 3T3 fibroblasts upon siRNA knockdown of mDia1, normalized per replicate to the mean cortical tension of cells transfected with scrambled siRNA (non-targeting); n_Cells_: -iMC+scrambled=31, -iMC+mDia1-siRNA=32, +iMC+scrambled=31, +iMC+mDia1-siRNA=37; n_Exp_: -iMC=3, +iMC=3. **d** Western blot comparing mDia1 expression upon transfection with non-targeting scrambled siRNA and mDia1-targeting siRNA, GAPDH was used as loading control. **e** Quantification of mDia1 protein upon siRNA knockdown of mDia1 based on the signal detected by western blot; the signal for each lane was normalized to the respective GAPDH signal, protein expression is shown relative to scrambled siRNA treated cells; dots show independent experiments, n_Exp_ = 3. **f** Schematic representation of domain structure of the two CAmDia1 constructs. A point mutation in the DAD domain (indicated by red stars) rendered the full-length construct, CAmDia1-FL, constitutively active. The truncated CAmDia1-ΔΝ was targeted to the membrane by an N-terminal lipidation motif. **g** Magnification of filopodia-like protrusions induced by expression of CAmDia1-ΔN or CAmDia1-FL; dashed white line marks the cell cortex. **h** Representative maximum intensity projections of fixed +iMC, +CAezrin and +iMC-6FP cells, co-expressing either CAmDia1-ΔN or CAmDia1-FL. **i** Filopodia number per cell, normalized to the mean filopodia number of cells expressing mDia1-ΔΝ, dots represent individual cells; n_Cells_: +iMC+CAmDia1-ΔΝ=58, +iMC+CAmDia1-FL = 63, +CAezrin+CAmDia1-ΔΝ=81, +CAezrin+CAmDia1-FL = 90, +6FP+CAmDia1-ΔΝ=76, +6FP+CAmDia1-FL = 64; n_Exp_: +iMC=2, +CAezrin=3, +iMC-6FP = 2. In all plots, bars show the mean and error bars the standard deviation. If not stated otherwise, dots in represent the mean of multiple measurements per single cell. n_Cells_= number of analysed cells of each condition, n_Exp_= number of independent experiments. Normality of data distribution was tested by Shapiro-Wilk test. Two-tailed t-test was used for normally distributed data. Otherwise, a two-sided non-parametric Wilcox test was used. See also Supplementary Figs. 8 and 9.

To decipher whether increased membrane-to-cortex attachment with a concomitant reduction in the membrane-to-cortex distance affects the activity of either of these actin nucleators, we first assessed cortical tension following inhibitor treatments in control and +iMC cells. Both an increase and a decrease in cortical tension upon Arp2/3 inhibition have been reported in the literature^9,53,54^. Inhibiting the Arp2/3 complex in interphase fibroblasts with the small molecule CK-666^55^ led to a minor increase in cortical tension in control cells (Supplementary Fig. 8a), which failed to mimic the mechanical impact of increased membrane-to-cortex attachment. This minor effect on cortical mechanics suggests that Arp2/3 activity is minimal in these cells, consistent with the cryo-ET data showing low abundance of actin branches in the cortex (Supplementary Fig. 5c and Supplementary Fig. 7b). Next, we performed similar experiments using SMIFH2, a pan-formin inhibitor^56^. In agreement with previous reports^57^, cortical tension was strongly reduced upon inhibitor treatment in our control cells, to levels similar to those measured in cells expressing the iMC-linker alone (Fig. 4a). Importantly, in cells expressing the iMC-linker, formin inhibition had no additional effect on cortical tension, suggesting that the iMC-linker acts upstream of formins in the reduction of tension. To rule out off-target effects of SMIFH2, which has been shown to inhibit members of the myosin superfamily at higher concentration than that used in this study^58^, we also tested the effect of direct myosin-2 inhibition. Blebbistatin treatment decreased cortical tension in both control and iMC-linker-expressing cells (Supplementary Fig. 8b), suggesting that our findings are not due to SMIFH2-mediated changes in myosin-2 activity. Next, to ensure that our measurements are not limited by the resolution limit of our setup we performed simultaneous inhibition of both cortical nucleators. This led to a strong reduction in cortical tension, irrespective of the level of tethering (Supplementary Fig. 8c). Finally, to test if the reduction in cortical tension by decreased membrane-to-cortex distance can be restored by activating formins, we used the small molecule IMM-01, which locks formins in their active conformation^59^. This treatment increased cortical tension in control cells, but failed to do so in cells expressing the iMC-linker (Fig. 4b). This strongly suggests that decreasing the membrane-to-cortex distance limits formin activity at the cell surface.

mDia1 has been shown to be the primary formin at the cortex^6^. First, we ruled out an effect of the iMC-linker on the expression of mDia1 by quantifying its levels by western blot (Supplementary Fig. 8d, e). Next, as an orthogonal and more specific approach to formin activity inhibition by SMIFH2, we designed and employed an siRNA to knock down mDia1 levels in control and +iMC cells (Fig. 4d, e and Supplementary Fig. 8f). Consistent with the results of our pharmacological panel, mDia1 knockdown decreased cortical tension in control cells, but had no effect in cells expressing the iMC-linker, indicating that there is a minimal level of basal mDia1 activity in +iMC cells (Fig. 4c). Combined with the cryo-ET results, we thus propose that reducing the size of the membrane-to-cortex gap causes a reduction in cortical tension via mDia1 inhibition. Of note, mDia’s activity is mechanosensitive and dependent on the environment^60,61^, being more processive when bound to the plasma membrane and upon pulling force on the polymerizing actin filament. In support of our hypothesis, negative stain electron microscopy structures indicate that formins undergo a conformational extension upon activation^62^.

If narrowing of the membrane-to-cortex gap inhibits mDia1 activity, we hypothesized that we could tune this effect by changing the relative size of mDia1 compared to the gap. To test this, we compared two GFP-tagged constitutively active mDia1 (CAmDia1) constructs that differ in molecular size (Fig. 4f), and expressed them in cells with various membrane-to-cortex distances (i.e., via expression of our linkers, see Fig. 3a, c).

The first construct, CAmDia1-FL^63^, was a full-length mDia1 variant containing a point mutation in its DAD domain, resulting in a constitutively active protein. The second construct, CAmDia1-ΔN, was a substantially smaller, truncated version, which lacks the N-terminal regulatory domains as well as the autoinhibitory DAD domain, retaining a minimal functional protein comprising the FH1 and FH2 domains^64^ required for actin polymerization. To ensure membrane targeting, CAmDia1-ΔN was fused to an N-terminal lyn motif (Fig. 4f).

Before co-expressing these CAmDia1 variants with our artificial linkers, we first examined their localisation and functionality in wild type cells. We observed that expression of either CAmDia1 construct triggered a dramatic morphological change: while cells without expression of CAmDia1 are marked by membrane ruffles and lamellipodial-like protrusions exclusively at the cell basal side (i.e., inset in Fig. 1a), cells expressing either CAmDia1 construct were covered in filopodia-like protrusions with CAmDia1 molecules concentrated at the tip (Fig. 4g, Supplementary Fig. 9a). This is consistent with the established role of formins in the nucleation of filopodia and with previous studies showing that mDia1 overexpression results in filopodia protrusion^65,66^.

Localisation and function of the two constructs was not altered by co-expression of an mCherry tagged lyn motif (Supplementary Fig. 9a), a control we used to exclude possible effects owing to the membrane targeting domain employed in our artificial linkers as well as the CAmDia1-ΔN. As a quantitative readout for mDia1 activity, we counted filopodia number using a costume-built pipeline based on automated spot detection (Supplementary Fig. 9b and compared the truncated (CAmDia1-ΔN) and the full-length (CAmDia1-FL) CAmDia1 constructs side by side. In control conditions (wild type and Lyn), both CAmDia1 constructs produced nearly equal number of filopodia (Supplementary Fig. 9c), indicating comparable activity.

We next co-expressed the CAmDia1 constructs together with CAezrin, the iMC-linker, or the iMC-6FP-linker to investigate how differences in the membrane-to-cortex gap affect mDia1 activity. Notably, in all conditions, both CAmDia1 constructs localised to the cell periphery, suggesting that neither was sterically excluded from the membrane-to-cortex interface (Supplementary Fig. 9d).

However, the quantification of filopodia number revealed a clear correlation between the size of the membrane-to-cortex gap and the activity of the differently sized CAmDia1 constructs (Fig. 4h, i): In +iMC cells, the larger CAmDia1-FL produced significantly less filopodia compared to the smaller CAmDia1-ΔN. This effect was also present, albeit to a lesser extent, in +CAezrin cells, and absent in +iMC-6FP cells, where both CAmDia1 constructs produced nearly equal number of filopodia. These results align well with our cryo-ET data, where iMC-linker expression leads to the strongest reduction in the membrane-to-cortex gap, followed by CAezrin, and with no effect for iMC-6FP-linker (Fig. 3c). Together, these results suggest that the smaller CAmDia1-ΔN construct retains its activity even when confronted with a narrowed membrane-to-cortex gap, whereas full-length CAmDia1-FL is physically inhibited in these conditions.

**Fig. 5:**
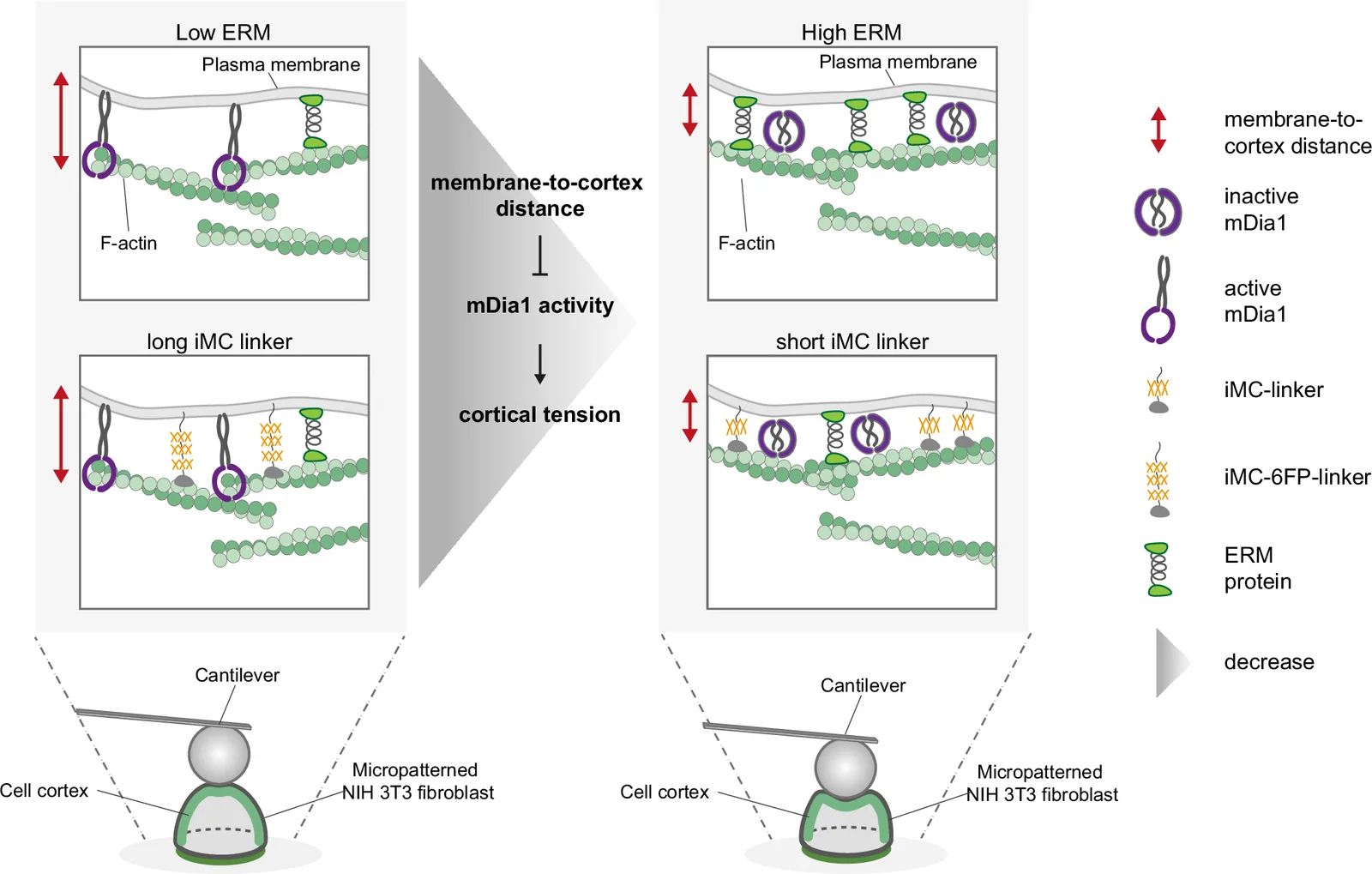
Proposed model for the regulation of cortical mechanics via the membrane-to-cortex distance. Our work identifies the membrane-to-cortex distance as an important parameter which contributes to the regulation of cortical mechanics. The dimension of this gap is regulated by the density and the length of membrane-to-cortex attachment proteins. The membrane-to-cortex gap regulates the function, though not the cortical localisation, of the formin mDia1 (represented by mDia1 in an extended, active conformation (left), or in a folded, inactive confirmation (right)). The consequent reduction in mDia1-mediated actin nucleation leads to a reduction in cortical tension.

In summary, we have shown that membrane-to-cortex linker proteins can narrow the gap between the membrane and the cortex in a length- and density-dependent manner. mDia1 is sensitive to the size of this gap, and its activity is inhibited below a critical gap size, leading to changes in cortical tension. Thus, the geometrical relationship between the size of mDia1 and the size of the gap between the membrane and the cortex controls mDia1 activity, offering a purely geometrical mechanism for the control of cortical mechanics (Fig. 5).

## Discussion

Membrane-to-cortex attachment has gained attention in recent years owing to its important role in the regulation of fundamental cellular processes such as stem cell differentiation^30,67^, cell division^25,26^ and migration^68^. However, the mechanisms by which it regulates such a diversity of cellular states have remained unclear. Interestingly, the spatial localisation of membrane-to-cortex attachment proteins often mirrors local differences in cortex mechanics, as seen for example in the apical domain of epithelial cells^29^ and early mouse blastomeres^28^, the back of migrating cells^24^, or the cytokinetic ring^25,26^, among others^69,70^. This raises the possibility that ERM proteins contribute to mechanically patterning cells.

In this study, we show that a seemingly simple geometrical parameter, the nanometer-scale membrane-to-cortex gap, regulates cell-scale mechanics at the cell surface. Specifically, we show that the density and length of membrane-to-cortex attachment proteins both contribute to determining the magnitude of the gap. Overexpression of a constitutively-active version of ezrin, as well as of a short artificial linker (iMC-linker), resulted in a reduction of the membrane-to-cortex distance (Fig. 2), and inhibited the activity of formin, an actin nucleator important for the generation of cortical filaments and filopodia (Fig. 4h, i). However, this effect is length-dependent. Overexpression of linkers just slightly longer than endogenous ERM proteins (*i.e*., the iMC-6FP-linker), neither changed the membrane-to-cortex distance nor inhibited formin activity (Fig. 3c, d and Fig. 4h, i).

If both length and density are relevant parameters, the question then arises as to how much diversity in length there is among endogenous membrane-to-cortex attachment proteins. The myosin-1 family and ERM proteins are canonical membrane-to-cortex linkers. Upon their depletion, a decrease in membrane tension has been measured by tether pulling approaches^68,71^. In some cases, such as for ezrin, the protein dimensions are known (25 nm^72^), and for the others, we provide AlphaFold2^73^ predictions (Supplementary Fig. 10a). In addition, we have previously found that depletion of the BAR-domain protein Snx33 leads to a reduction in membrane-to-cortex attachment, identifying it as non-canonical membrane-to-cortex attachment protein (Supplementary Fig. 10b). This underscores that linker proteins are likely not restricted to a few archetypical families; any protein or protein complex with both actin and membrane-binding domains could potentially function as a membrane-to-cortex attachment protein. Thus, future studies will be needed to reveal the true repertoire of membrane-to-cortex attachment protein lengths available to cells.

A functional role for the density of linker proteins has been proposed in the context of cell migration^74,75^, where a decrease in membrane-to-cortex attachment acts as a break-release mechanism, allowing lamellipodia initiation. Welf et al. elegantly incorporate ERM proteins into the classic tethered ratchet model^76^. In their model, membrane-to-cortex attachment acts as a force dissipator, preventing polymerizing actin filaments from pushing against the plasma membrane. As a consequence, actin-membrane detachment is an essential prerequisite for protrusion initiation. The length of membrane-to-cortex attachment proteins remains a less explored parameter, partly due to the lack of tools to enable its study in cells. In light of our findings, it would be interesting to test if the expression of a longer linker, such as the iMC-6FP-linker, would still prevent actin polymerization in migrating cells.

Further, Bisaria et al. describe a cofilin-driven back-to-front gradient in actin next to the plasma membrane (the so-called membrane proximal F-actin). This gradient allows Rac1 activity to proceed at the front of migrating cells. However, how proximal actin impairs Rac1 activity remains unexplored. We show that the membrane-to-cortex distance is a functional geometrical parameter in cells that directly regulates mDia1 activity. Perhaps future studies could explore whether a similar mechanism can be invoked to explain the regulation of other molecular machines, such as Rac1, that act at the cell periphery.

The inhibition of formin activity that we observe could result from the inability of formins to effectively assume their active extended conformation, or from the failure of formins to elongate filaments efficiently. Interestingly, the sharp transition between the ~25-nm CAezrin^72^/iMC-4FP-linker and the ~30 nm iMC-6FP-linker is relatively consistent with the predicted structure of active formins, which are proposed to extend to around 20 nm in the active confirmation^62^. Our observation of the same mechanical phenotype in iMC-linker- and CAezrin-expressing cells supports an unspecific inhibition of a geometrical nature. Our experiments knocking down endogenous mDia1 allow us to identify the formin at play (Fig. 4c). Finally, the use of constitutively active mDia1 constructs of different lengths, shows that the membrane-to-cortex distance by itself directly regulates mDia1’s ability to polymerize actin (Fig. 4h, i). Given the mechanosensitivity of formins^77–80^, specifically that of the key formin at the cell cortex mDia1^6,53,78,79,81^, it is tempting to speculate that a feed-forward loop might be at play: membrane-to-cortex attachment may alter formins’ ability to efficiently elongate filament ends^81^ at the cell surface in the first place, which would lead to a reduction in cortical bundles and tension. As formins are more processive upon tension on the polymerized filament^79,80^, this further decreases formin-dependent filament elongation.

In this paper, we provide the first characterization of cortical architecture in native cell cortices away from the adhesive substrate, and do so with single-filament resolution. Connecting cell-scale mechanics to defined cytoskeletal topologies is a current challenge in mechanobiology. There is a need to develop methods that allow descriptions of cytoskeletal network architectures in quantitative terms at subcellular resolution, to guide reconstitution and modelling efforts, and interpret biophysical measurements. Although other actin network structures have been resolved by cryo-ET^42,82–84^, these studies have so far been limited in scope to particular cell states (such as mitotic cells) and very specific cellular regions such as lamellipodia (which can be imaged without the need for milling due to their limited thickness). In this study, we show that interphase cortices away from the adhesive substrate are locally highly diverse in both architecture and density (Figs. 2 and 3, Supplementary Fig. 3 and Supplementary Fig. 4), with relatively few occasions of actin branching (Supplementary Fig. 5c and Supplementary Fig. 7c. The surface of cells, imaged in previous SEM and atomic force microscopy studies, sometimes appears to exhibit densely connected networks^5,85,86^, but also sparse surfaces^16,87^. In addition, such imaging approaches provide insights with lower resolution (Scale bar = 1 µm in Kronlage et al.^85^) compared to cryo-ET, and often cannot distinguish intermediate and actin filaments. Our approach provides single-filament resolution and cytoskeletal identity, unachievable with other methods. However, cryo-ET comes with the drawback of visualising only a small volume of large cells. Hopefully future advances in super-resolution light microscopy will move towards single-filament resolution at a whole-cell scale. This would allow us to reconcile the high level of variability seen among studies and facilitate a more complete understanding of how cells regulate the mechanical and geometrical properties of their cortices in different states.

A key aspect of our characterization is the statistical description of the membrane-to-cortex distance at the nanometer-scale. We show that actin filaments are very infrequently observed within 15 nm of the membrane, a key distance for proteins active at the surface. In fact, previous attempts to quantify this gap from 2D images by stimulated emission depletion (STED) microscopy in live cells found a bimodal distribution of this separation within a single cell, suggesting that some regions of the membrane are closer to the actin cytoskeleton than others^18^. Going forward, it would be interesting to see if these areas are endowed with higher linker density, or a different set of linker proteins.

At the cell periphery, patterning of protein activities leads to differential mechanics^88,89^, but the organizing principles behind this are in general poorly understood. Here, we link cell-scale mechanics to its molecular constituents in 3D and identify the membrane-to-cortex distance as a control switch for formin activity. Given the widespread roles of membrane-to-cortex attachment proteins in processes ranging from differentiation to migration, our work raises the intriguing idea that the membrane-to-cortex gap could regulate multiple molecular machines at the cell periphery. Importantly, the mechanism we have identified is inherently robust due to its reliance on geometrical constraints rather than precise intermolecular interactions. Thus, we propose that by regulating the expression and localisation of membrane-to-cortex attachment proteins – which are evolutionary conserved throughout metazoa^90^ – cells can control this key geometrical parameter to pattern myriad biological processes.

## Limitations of the study

For this paper, we developed and employed new techniques which were essential to be able to disentangle the multiple complex parameters at the cell surface and draw robust mechanistic conclusions. In particular, we employed our signalling-inert artificial linkers, as well as constitutively active ezrin and mDia1. However, our paper stops short of demonstrating that this mechanism is at work in an unperturbed biological system. This is a key limitation of our work. Nonetheless, as discussed in the previous paragraphs, the internal landscape of the cell is well equipped to put this mechanism into practice, and there are exciting hints in the literature which suggest physiological processes where our mechanism may be at work.

## Methods

### Cell culture

NIH 3T3 fibroblasts (CRL-1658, ATCC repository) were cultured in growth medium (Dulbecco’s Modified Eagle Medium (DMEM) with 4.5 g/L D-glucose (Merck), supplemented with 10% fetal bovine serum (FBS, #A5256701, Thermo Fisher Scientific) and 1% Penicillin-Streptomycin (10,000 U/mL, #15140-122, Gibco). Mouse Embryonic Stem Cells (mESCs, kindly provided by Austin Smith’s group, Cambridge Stem Cell Institute), were maintained in serum-free N2B27 medium containing 2i (1 mM PD0325901, #4192 and 3 mM CHIR99021, #4423, both Tocris) and LIF (10 mg/mL, EMBL protein expression facility) on polystyrene culture dishes coated with 0.1% (w/v) Gelatin (#ES-006-B, Sigma-Aldrich) solution. N2B27 medium was prepared from a 1:1 mixture of DMEM/F12 (without HEPES, with L-glutamine, #11320033, Thermo Fisher Scientific) and neurobasal medium (no L-glutamine, #21103049, Thermo Fisher Scientific), supplemented with 0.5x B-27 (without vitamin A, # 12587010, Thermo Fisher Scientific) and 0.5x N-2 supplement (#17502-048, Thermo Fisher Scientific), 2.5 mM L-glutamine (#A2916801, Gibco), 10 mg/mL BSA fraction (#A7030, Sigma-Aldrich), 10 mg/mL human recombinant insulin (#91077C, Sigma-Aldrich) and 1% Penicillin-Streptomycin (10,000 U/mL). WT and Snx33 KO HL-60 cells^91^ were grown in RPMI 1640 media (#10-040-CV, Corning) with 10% heat-inactivated FBS (#10500-064, Gibco) and 1% Penicillin-Streptomycin; cells were differentiated by adding 1.5% DMSO (#D2438, Sigma-Aldrich) and used after 5 days. All the cells were cultured at 37 °C with 5% CO_2_ on polystyrene 100-mm dishes. NIH 3T3 fibroblasts and mESCs were passaged using 0.05% Trypsin-EDTA (#25300-054, Gibco). Cells were routinely tested for mycoplasma contamination using MycoAlert detection kit (#LT07, Lonza) according to manufacturer’s instruction and using the Mycoplasmacheck Service from Eurofins Genomics.

### Cell line generation

Transgenic cell lines used in this study were generated using a PiggyBac transposon system^92^ or lentiviral transduction.

For cell line generation with the PiggyBac transposon system, the gene of interest was cloned via Gibson assembly into a custom PiggyBac vector downstream of a doxycycline (dox)-responsive TRE3G promoter. The transposable region of the vector also contained a TET3G and a Neomycin resistance cassette under the control of a CAG promoter. Stable cell lines were obtained by co-transfecting the PiggyBac vector and the PiggyBac transposase using Lipofectamine 3000 (#L3000-008, Invitrogen). Successfully transfected cells were then positively selected by adding 1.2 mg/mL Geneticin (#10131-035, Gibco) to the medium. Expression of the constructs was induced by the addition of 1 µg/mL doxycycline (#D9891, Sigma-Aldrich) to the medium 16 hrs before experiments.

For lentiviral transduction, infectious particles were produced in HEK-293T cells. Cells at ~60% confluency were transfected with 1.5 μg of transfer plasmid (containing the gene of interest), 0.9 μg of the viral envelope plasmid (pMD2.G, #12259, Addgene) and 2.7 μg of the viral packaging plasmid (pCMV 8.74, #22036, Addgene). Transfection was performed using the TransIT-Lenti reagent (#MIR 6603, Mirus) together with OptiMEM (#31985070, Gibco) according to the manufacturer’s protocol. Infectious viral particles were harvested on day two post-transfection and concentrated 25-30x by using Lenti-X concentrator (#631231, Takara). In brief, the supernatant of transfected HEK-293T cells was filtered through a 0.22 µm PVDF filter and mixed with 1/3 volume of the Lenti-X concentrator. After incubation at 4 °C for 30 min, the mixture was centrifuged at 1,500 g at 4 °C for 45 min. The resulting pellet was resuspended in 50–100 µL of cell culture medium to infect target cells. Target cells were treated with 5 µg/mL polybrene (#TR-1003-G, Merck) shortly before infection.

Monoclonal cell lines were generated by limiting dilution, and screened via flow cytometry (BD LSRFortessa, BD FACSymphony) for low background fluorescence. In brief, cells were detached, and single cells were strained through a 70 µm pore size cell strainer and suspended in flow buffer (PBS supplemented with 0.5% BSA, 2.5 mM EDTA). As the sample originated from a pure culture, cells were gated with a simple gating strategy using FCS/SSC to remove doublets and debris. To ensure consistency in expression levels of constructs upon dox-induction, cell lines with a geometric mean at least 30 times higher than that of the background fluorescence of the respective control cells were used for experiments (matching the dashed line in Fig. 1d).

### Construct generation

All constructs used in this study are listed in Supplementary Table 2.

The construct of the iMC-6FP-linker and iMC-4FP-linker were generated by insertion of non-fluorescent mCherry proteins (dark mCherry, Y72S; 5x dark mCherry for iMC-6FP, 3x dark mCherry for iMC-4FP) between the fluorescent mCherry molecule and the actin binding domain of the iMC-linker^45^. The sequence for dark mCherry molecules connected by an Ala-Ala-Gly-Gly-Gly-Gly-Ser-Thr-Ser spacer was designed and purchased from GenScript. For cloning purposes, the two alanine were removed in every second spacer. Single dark mCherry molecules were added using Type IIS endonuclease cloning^93^. The length of the molecule was predicted based on the crystal structure of a single mCherry molecule^94^. The construct of CAmDia1-ΔΝ was generated based on CAmDia1-FL^63^.The sequence between position 543 and 1192 of the full-length protein was amplified by PCR using the forward primer gctgtacaagtccggactcagatctTCAAAAGAACTAGAAGATGCCAAG and reverse primer atcccgggcccgcggtaccgtcgactgcggccgcagcttaCCCTGACTGCAGAGCTTC and inserted via Gibson assembly into the transfection backbone. In a second step an N-terminal lyn lipidation motif was added before the GFP tag via Gibson assembly, using the forward primer GAATTCAcgcgtgccaccatgggatgtataaaatcaaaagggaaagacagcgcgggaGTGAGCAAGGGCGAGGAG and reverse primer ttcatcgcGGCCGCttcatcttaCCCTGACTGCAGAGCTTC.

### Cellular micropatterning on glass

NIH 3T3 fibroblasts were seeded on 20 µm adhesive spots to ensure a spherical shape and uniform cortex. For micropatterning of the dishes, an inverted Ti2^®^ Nikon microscope equipped with the Primo micropatterning module (Alvéole) was used. In brief, 35 mm glass-bottom dishes (#627860, Greiner bio-one, FluoroDish #FD35-100, World Precision Instruments) were plasma treated for 1 min, and a small PDMS stencil with an inner hole of ~10–20 µL was placed in the middle of the dish to reduce reagent usage. Next, the glass surface inside the stencil was coated with 0.2 mg/mL polyethylene glycol (PLL(20)-g[3.5]- PEG(2), PLLg-PEG, Susos AG) for 1 hour at room temperature (RT) to form a non-adhesive layer. Then, the surface was washed thrice with PBS before applying a thin layer of 4-benzoylbenzyl-trimethylammonium chloride micropatterning reagent (PLPP, Alvéole). Using a 375 nm laser (4.5 mW), 20 µm circles spaced by 40 µm were photo-patterned onto the glass surface, using the µmanager software v.1.4.22 with the Leonardo plugin v.4.12 (Alvéole). Finally, the dish was coated with 50 µg/mL human fibronectin (#356008, Corning) for 30 min and 10^5^ cells were seeded onto the patterned dish. Cells were allowed to settle and adhere for 20 min before non-attached cells were washed out. Experiments were performed 2 hrs after cell seeding.

### Single-cell atomic force spectroscopy

Tether extrusion and nano-indentation were performed on a JPK CellHesion^®^ 200 (v.6) atomic force spectrometer (Bruker) integrated into an Eclipse Ti^®^ inverted light microscope (Nikon). Measurements were run at 37 °C with 5% CO_2_ and samples were used no longer than 1 h for data acquisition. Measurements were acquired at a sampling rate of 2 KHz and analysed using the JPK SPM Desktop and Processing Software (version 6.4.39).

Apparent plasma membrane tension (*T*_*(app)*_) (the sum of in-plane tension (*T*_*m*_) and the adhesion energy resulting from membrane-to-cortex attachment (*γ)*) depends on the breakage tether force (*f*_*0*_) and the bending rigidity of the membrane (*κ*)^95^, which we assumed is unchanged between our experimental conditions.

1 $$\begin{array}{c}{T}_{\left({app}\right)}={T}_{m}+\gamma=\frac{{f}_{0}^{2}}{8{\pi }^{2}\kappa }\end{array}$$

To measure the breakage tether force, we used OBL-10 Cantilevers (spring constant ~0.06 N/m; Bruker) calibrated using the thermal noise method (reviewed in ref. ^96^) and coated for 1 h at 37 °C with 2.5 mg/mL Concanavalin A (#C5275, Sigma-Aldrich), which binds polysaccharides expressed on the surface of the cell. Before the measurements, cantilevers were rinsed in PBS. NIH 3T3 fibroblasts were washed and probed in DMEM with 4.5 g/L D-glucose supplemented with 2% FBS and 1% Penicillin-Streptomycin (10,000 U/mL), mESCs were probed in growth medium (described above in the Cell culture section).

In brief, approach velocity was set to 0.5 μm/s while contact force and contact time ranged from 100 to 200 pN and 100 ms to 10 s, respectively, in order to maximize the probability to extrude single tethers, while minimizing the experimental stress on the cells. After contacting the cell surface, the cantilever was retracted for 10 μm at a velocity of 10 μm/s. The position was then kept constant for ~30 s and the tether force was recorded at the moment of tether breakage. Each cell was probed for a maximum of 10 attempts or until 3 single tethers were successfully extruded. Tether force values from a single cell were then averaged.

Dynamic tether pulling was deployed to measure the contribution of Snx33 to membrane-to-cortex attachment. Plasma membrane tethers were pulled at different retraction velocities (*v*) (2, 5, 10 and 30 µm/s) and the tether force (*f*) was measured during retraction. In order to extrapolate the membrane-to-cortex attachment parameter (*a*), the Brochard-Wyart et al. model was applied to the data using Monte-Carlo based fitting^97^.

2 $$\begin{array}{c}{f}^{3}-f{f}_{0}^{2}={av}=\left[{(2{{\rm{\pi }}})}^{3}2{\kappa }^{2}\eta \varepsilon {\mathrm{ln}}\left(\frac{{R}_{c}}{{R}_{t}}\right)\right]v\end{array}$$

where the radius of the cell (*R*_*c*_) is >> the radius of the tether (*R*_*t*_), and the bending rigidity (κ) is assumed to be constant. Therefore, the increase in tether force upon increasing pulling velocity depends exclusively on the surface viscosity (*η*) and the density of the membrane-to-cortex attachment linkers (*ε*). The term *ηε* is proportional to the membrane-to-cortex attachment parameter *a*, used here as a proxy for membrane-to-cortex attachment.

For cortical tension measurements on adherent cells, nano-indentation was performed using tipless MLCT-O10 type C cantilevers (spring constant ~0.01 N/m; Bruker), calibrated using the thermal noise method^96^. A silica bead with a diameter of 10 μm (Microparticles) was glued onto the cantilever using a 2-part epoxy resin (UHU) with 5 min working time. Upon full hardening of the glue, the cantilever was coated for 30 min at 37 °C with a 1% (w/v) Pluronic F-127 (#P2443, Sigma-Aldrich) in milli-Q water and rinsed with PBS, aiming to prevent non-specific adhesion to the probed cells. Force-distance curves were acquired using 500 pN contact force and 0.4 μm/s approach/retract velocity. Up to 5 curves were taken per cell, with a 10 s waiting time between successive curves to prevent history effects.

Cortical tension (*T*_*c*_) was determined by fitting each force-indentation curve between 0 (the contact point) and 300 nm with a liquid droplet model suited for nano-indentation experiments^98^.

3 $$\begin{array}{c}f=\left[2{T}_{c}\left(\frac{1}{{R}_{c}}+\frac{1}{{R}_{b}}\right)\times 2\pi {R}_{b}\right]\times \delta \end{array}$$

Where *f* is the force applied to the cell surface which leads to a displacement (δ) of cellular material; *R*_*c*_ and *R*_*b*_ are the radius of the cell and the silica bead glued on the cantilever, respectively.

### Micropipette aspiration

Micropipettes were forged by pulling glass capillaries (TW100-3, World Precision Instruments) using a P-97 Flaming Brown needle puller (Sutter Instrument) in order to obtain a radius (*R*_*p*_) of 4–6 μm. The micropipette was mounted on a micromanipulator (MON202-D, Narishige) and connected to a microfluidic pump (Fluigent, MFCS) delivering negative pressures with a 7 Pa resolution. The pressure was controlled using a custom-made LabVIEW (National Instruments) software.

To measure cortical tension, NIH 3T3 fibroblasts were detached from the substrate using 0.05% Trypsin-EDTA, seeded on a 50 mm glass-bottom dish pre-coated with a non-adhesive layer of 0.2 mg/mL PLLg-PEG, and incubated at 37 °C with 5% CO_2_ for 4 h to allow cell shape recovery. After the incubation, cells were probed by bringing the micropipette in contact, and increasing stepwise a negative pressure until a deformation of the cells with the same radius of the micropipette (*R*_*p*_) was formed. At steady state, the surface tension (*T*_*c*_) of the cells was calculated based on Young–Laplace’s law^99^.

4 $$\begin{array}{c}{T}_{c}=\left[\frac{{P}_{c}}{2}\left(\frac{1}{{R}_{p}}-\frac{1}{{R}_{c}}\right)\right]\end{array}$$

Where *P*_*c*_ is the pressure used to deform the cell of radius (*R*_*c*_).

### Drug treatments

NIH 3T3 fibroblasts were seeded on micropatterned dishes as described above and actomyosin perturbing drugs were added directly to the medium to reach a final concentration of 40 μM for SMIFH2 (#S4826, Sigma-Aldrich), 100 μM for CK-666 (#3950, Tocris), 25 μM for IMM-01 (#SML1064, Sigma-Aldrich) and 20 μM for (-)-Blebbistatin (#B0560, Sigma-Aldrich). Cells were then incubated with the drug for 1 h before probing with an atomic force spectrometer in presence of the drug. Control cells were treated with vehicle only (DMSO).

### Magnetic pincher

24–48 h before magnetic pinching experiments, NIH 3T3 fibroblasts were seeded at a density of 5*10^3^ cells/cm^2^ in the presence of superparamagnetic beads (M-450, Dynabeads) at a density of 10^4^ beads/cm^2^.

On the day of the experiment, cells were detached from the substrate using 0.05% Trypsin-EDTA and plated on micropatterned dishes as described above. Then, 1.5*10^5^ M450 beads were added to the dish, and the medium was supplemented with 20 mM of HEPES buffer, to mitigate pH fluctuations during the experiment.

The magnetic pincher setup was mounted on an Axio A1 inverted microscope (Zeiss) with an oil-immersion 100x objective mounted on a piezo-controlled translator (Physik Instrumente). The magnetic field was generated by two coaxial coils (SBEA) with mu metal core (length = 40 mm; diameter = 26–88 mm; 750 spires). The coils were powered by a bipolar operational power supply amplifier 6 A/36 V (Kepco) controlled by a data acquisition module (National Instruments). The maximum field generated was 54 mT with a gradient less than 0.1 mT/mm over the sample. The self-organization of beads was first triggered with a constant field of 5 mT. Time-lapse images were recorded with an Orca Flash4 camera (Hamamatsu Photonics). The setup was heated at 37 °C, without CO_2_ supplementation and controlled by a custom LabVIEW (National Instruments) interface to ensure the synchronicity between piezo position, magnetic field imposition, and image acquisition.

Cells with their cortex pinched between beads were imaged. For each video, the nominal magnetic field exerted by the coils was 5 mT (108 pN) and every 10 s the field was lowered to 1 mT (25 pN), then increased to 54 mT (1090 pN) in 1.5 s, then brought back to 5 mT (the values between brackets are the typical corresponding force values). This series of compression-relaxation was repeated 6 to 10 times per cell. In addition to the images, the precise time and magnetic field corresponding to each image were saved. Using Fiji’s plugin “Analyze Particle”, the centre of all beads on each image of the time-lapse was detected with subpixel resolution^37^. Then using a custom-made tracking algorithm written in Python (https://github.com/jvermeil-biophys/CortExplore_PublicVersion) the trajectories in 3D of the centres of the two beads pinching the cortex were detected, and the thickness of the pinched cortex was computed. The pinching force was also computed knowing the distance between the beads, the external magnetic field, and the magnetization function of the beads. To characterize the cortex thickness, the median of the cortical thickness at 5 mT (nominal field) was measured; it corresponds to a typical force of 108 ± 29 pN. The elastic modulus at low stress was computed from the strain-stress curve corresponding to each compression using a model developed by Chadwick for this specific geometry^100^. Next, the slope of these strain-stress curves between 100 and 300 Pa was fitted.

### Immunofluorescence staining and quantification

For the staining of p-myosin and actin in fixed cells, cells were seeded into micropatterned 12-well glass bottom plates, fixed and permeabilized simultaneously by adding prewarmed (37 °C) fixation buffer (2x) to growth medium, and incubated for 20 min at RT. Fixation buffer (1x) contains 4% PFA (#28908, Thermo Fisher Scientific), 0.5% Tergitol (#37242.01, Serva), 20 mM sucrose (#S0389, Sigma-Aldrich) in cytoskeleton buffer (10 mM MES, 140 mM KCl, 3 mM MgCl_2_, 2 mM EGTA, pH = 7.5). Cells were washed thrice for 10 min with 0.05% Tergitol in TBS (Tris-Buffered Saline) and blocked for 1 hour with 2% BSA (#A7030, Sigma-Aldrich) in TBS. Primary antibody staining for p-myosin (anti-Phospho-MLC2 (Ser19), 1:100, #3671, Cell signalling technology), together with actin staining (AF647 Phalloidin,1:2,000, #A22287, Thermo Fisher Scientific) was performed overnight at 4 °C in blocking solution. Cells were washed thrice for 10 min with 0.05% Tergitol in TBS and incubated in blocking solution for 10 min. Secondary antibody staining was performed for 1 h at RT in blocking solution (AF488 goat-rabbit, 1:1,000, #A11034, Thermo Fisher Scientific). Cells were washed thrice for 10 min before imaging in TBS. Z-stacks of stained cells were acquired with 0.6 µm step size on a Ti-2^®^ CSU W1 SORA spinning disk (Nikon) with a 60x water immersion objective.

For the quantification of cortical actin in live cells, F-actin was visualized in micropatterned cells with the live actin dye SPY650-FastActTM (1:1,000, Spirochrome). Cells were stained in growth medium (see Cell culture section for details) for 2 h at 37 °C. As a negative stain used for segmentation, 0.01 µg of AF488 N-Hydroxysuccinimide (NHS) ester (#11820, Lumiprobe) was added to the medium shortly before imaging. Cells were imaged using an inverted confocal laser scanning microscope (Leica Stellaris 8^®^, HC PL APO CS2 40x / NA 1.10 water objective lens, white light laser) with the Leica Application Suite X 4.8.1.29271 (LAX) software. For each cell, one plane was recorded with a 4 µm set-off from the coverslip.

To analyse cortical myosin−2 in live cells, a myosin reporter was used^57^, encoding for the GFP-tagged light chain of myosin-2 with continuous expression. The myosin reporter was introduced into the clonal iMC-linker cell line via lentiviral transduction, and a single cell clone was selected to ensure consistent reporter expression. Cells were micropatterned and imaged in the presence of an AF647 NHS-ester (#16820, Lumiprobe) for negative staining. Z-stacks of stained cells were acquired with 0.3 µm step size at Ti-2^®^ CSU W1 SORA spinning disk (Nikon) with 60x water immersion objective with the NIS Elements AR 5.30.07 software.

Fluorescence intensities were analysed in ImageJ2 (version 2.9.0/1.54b) using a custom-made Fiji macro with the MorpholibJ plugin. In brief, in fixed cells, the F-actin stain was used to segment the cell boundaries; in live cells a negative stain was used. The cortical area was determined within a range of 0.6−1 µm from the segmentation boundary, for which the mean fluorescence signal of F-actin, p-myosin, myosin−2 and the iMC-linker was measured. Mean fluorescence intensities were normalized to the control for each replicate.

### mDia1 knockdown

For the siRNA-mediated knockdown of mDia1, a targeting siRNA was designed following well-established guidelines^101^. In brief, we first obtained the sequences of all the alternative transcripts derived from the *DIAPH1* gene in *Mus musculus* (C57BL/6 strain) using the ENSEMBL genome database. A region of 100% identity between the three protein-coding transcripts was identified within the second exon, and candidate siRNAs were designed with Block-iT RNAi designer (Thermo Fisher Scientific) online tool following stringent criteria (e.g., specific GG content and distribution, and absence of regions with long repeats). The siRNA candidate sequences were finally blasted against the C57BL/6 mouse strain transcriptome to assure minimal off-target effects.

The chosen mDia1 targeting siRNA (5’−3’ CUUCGAGUCUCUCUCAACAAU) was synthesized by Thermo Fisher Scientific and introduced into the cells via electroporation using the Neon transfection system (Thermo Fisher Scientific). Per experiment, two 10 µL transfection reactions were pooled. For each reaction 5*10^4^ cells in 10 µL Resuspension Buffer R were transfected with 0.4 µM siRNA using the following parameters (according to the manufacturer): Pulse Voltage: 1,400 V; Pulse Width: 20 ms; Pulse number: 2. After electroporation, cells were directly added into 2 mL prepared growth medium, which was exchanged once, 2 h after the treatment. Cells were used 3–4 days after knockdown for subsequent experiments. As a control the non-targeting “scrambled” siRNA (Silencer Select Negative Control #1 siRNA, #4390843, Thermo Fisher Scientific) was included. Successful knockdown was verified by western blot, for details see western blotting section below.

### Western blotting

For sample collection, cells were lysed in RIPA Lysis and Extraction buffer (#89900, Thermo Fisher Scientific) supplemented with protease inhibitors (#4693159001, Roche) according to manufactures’ protocol. After removal of cell growth medium, cells were washed twice with cold PBS (4 °C) before incubation in lysis buffer for 5 min on ice. Lysed cells were collected using a cell scraper. Cells were either sonicated for 2 min to enhance lysis, centrifuged for 15 min at 14,000 g at 4 °C and the collected supernatant was treated with Bezonase (1:100) and 0.5 M MgCl_2_ (1:100) for 10 min at RT. Alternatively, cells were directly centrifuged at 16,000 g for 15 min at 4 °C. For blotting, samples were denatured by mixing them with 4x Laemmli buffer (#161-0747, BioRad) containing 10% β-mercaptoethanol (m6250, Sigma-Aldrich) and incubated for 5 min at 95 °C. Proteins were separated in a SDS page using Nupage 4–18% Bis-Tris gels in running buffer (50 mM MOPS, 50 mM TrisBase, 3.5 mM Sodium dodecyl sulfate, 1 mM EDTA). Next, proteins were transferred by wet blotting in transfer buffer (25 mM TrisBase, 200 mM glycine, 20% methanol) on a methanol activated PDVF membrane. Successful protein transfer was verified by Ponceau red staining. For immunodetection, all washing steps were performed in TBS-T (TBS supplemented with 0.001% Tween). The blot was blocked in 5% BSA in TBS-T and subsequently incubated with primary antibody (anti-mDia1, 1:1,000, #20624-1-AP, Thermo Fischer Scientific; anti-phospho-Ezrin (Thr567)/Radixin (Thr564)/Moesin(Thr558), 1:500, #3141, Cell Signaling; GAPDH, 1:10,000, #NB300-221, Novus Biologicals) overnight at 4  °C. Secondary antibody staining was performed for 1 h at RT in blocking solution (Donkey-Anti-Rabbit-HRP, 1:20,000, #711-035-152, Jackson Immuno Research; Goat-Anti-Mouse-HRP, 1:10,000, #115-035-062, Jackson ImmunoResearch). Proteins were detected using ECL western blotting substrate and developed on an iBright Imaging Reader.

Western blots were quantified in Fiji. In brief, bands of interest were selected by the square tool and the integrated density of the target protein was normalized to that of the loading control lane. The mean of technical replicates was calculated and samples were normalized to control samples.

### Cryo-electron tomography

Micropatterns for cryo-electron tomography (cryo-ET) were designed as two 10-µm diameter circles with a separation of 15 µm per grid square. Micropatterns covered an area of 8 × 8 squares around the centre of the cryo-TEM grids (Quantifoil R1/4, R2/1 or R1/20, Au 200 mesh, SiO_2_ film)^41^. Grids were treated with plasma (Fischione instruments, model 1070 Nanoclean) on both sides for 2 min. Next, grids were incubated on droplets of poly-L-lysine-graft-polyethylene glycol (PLLg-PEG) at a concentration of 0.5 mg/mL in 10 mM HEPES pH 7.4 for 1 hour at RT on parafilm in a humid chamber. Following this passivation, the grids were blotted with filter paper from the back and immediately placed on a drop of PLPP. Grids were then photo-patterned using the Primo module with a 375 nm laser (4.5 mW) as described above. Grids were promptly retrieved from the PLPP solution, washed first in milli-Q water and then in PBS. For functionalization of the micropatterns, grids were incubated for 45 min at RT or overnight at 4 °C with bovine fibronectin in PBS. Grids were then stored in PBS at 4 °C in a humid chamber until use.

After overnight expression of the respective construct, NIH 3T3 fibroblasts were seeded at a concentration of 10^6^ cells/mL and allowed to settle and adhere for 1–2 h before non-attached cells were washed out. Subsequently, 3.5 µL of growth medium was applied to the frontside of the grids and a Leica EM GP2^®^ was utilized to vitrify cells. Blotting from the backside was performed for 3 s at 37 °C and 70% humidity. Grids were plunge-frozen in liquid ethane cooled by liquid nitrogen and stored until further usage.

For cryo-focused ion beam (cryo-FIB) milling, each grid was clipped into an autogrid cartridge with a cut-out enabling milling at shallow angles. Mounted on 35° or 45° pre-tilt shuttles, grids were transferred into an Aquilos Dual beam microscope (Thermo Fisher Scientific) operated with SerialFIB (version 1.0, github.com/sklumpe/SerialFIB) via a load-lock system and maintained at −183 °C throughout the next steps. Prior to cryo-FIB milling, a layer of organometallic platinum was applied by opening the gas injection system for 10 s at a stage Z position of 3–4 mm below the coincidence point. Subsequently, grids were sputter-coated with platinum for 20 s (1 kV, 10 mA, 10 Pa). In independent sessions, lamellae were prepared at milling angles ranging from 7 to 11°. Grid squares with two cells attached in the desired geometry were micromachined sequentially in three steps of rough milling to a thickness of 5 µm at 1 nA ion beam current, 3 µm at 0.5 nA and finally to 1 µm at 0.1 nA. Fine milling was performed at 100 and 50 pA to a target thickness of 200 nm. To render the lamellae conductive for cryo-ET imaging, they were sputtered with platinum for 3 s (1 kV, 10 mA, 10 Pa) and transferred into sealed boxes in liquid nitrogen.

For cryo-electron tomography, autogrids containing lamellae were loaded into a Titan Krios microscope (Thermo Fisher Scientific) such that the axis of the lamella pre-tilt introduced by cryo-FIB milling was aligned perpendicular to the tilt axis of the microscope. Tomograms were acquired at 300 kV on a K2 Summit direct detection camera (Gatan) operating in dose fractionation mode and utilizing a Quantum post-column energy filter operated at zero-loss (Gatan). Data were recorded using automated acquisition procedures in the SerialEM software (version 4.1.0bet, doi: 10.1016/j.jsb.2005.07.007)^102^. Magnification of 42,000 (EFTEM) with a calibrated pixel size of 3.37 Å was used for data collection. Starting from the lamella pre-tilt, images were acquired in 2° increments using a dose-symmetric tilt scheme^103^. A maximum of 125 e^-^/Å^2^ total dose was used for tilt-series acquisition with a constant electron dose per tilt image. Tilt-series were collected either with a Volta potential phase plate (VPP, Thermo Fisher Scientific) pre-conditioned for 5 min at a defocus range of −2 to −4 µm or without VPP at a defocus range of −6 to −8 µm. Cryo-electron tomography data analysed in this work are summarized in Supplementary Table 3.

For tomogram reconstruction, tilt movie frames were aligned using a SerialEM plugin or in Warp (version 1.0.7)^104^. Tilt series alignment was performed in the IMOD (version 4.9) software package^105^, using fiducials (platinum particle contaminants on the lamellae surface) or patch-tracking. Aligned images were binned to a pixel of 13.48 Å. Tomographic reconstruction was performed in IMOD. An amplitude spectrum matching filter was applied in DeePiCt (version 1.0.0)^106^ to all tomograms used for segmentation, including those shown in Fig. 2, Supplementary Figs. 3–6, and Supplementary Video 1.

Actin segmentation was performed with the automated filament tracing module in Amira^107^ (version 2022.1) using a generic missing wedge-modulated cylinder with a radius of 4 nm and 40 nm length as a template. The traced filaments were manually curated using Dragonfly software (version 2022.2, Comet Technologies Canada Inc.). The curated filaments were exported as a binary mask and the coordinates were resampled to equidistant points corresponding to the size of an actin monomer (approximately 4 nm) using a custom Python script. Briefly, the actin filaments were eroded and skeletonized, and any branch points after skeletonization were removed to avoid ambiguities in actin filament assignment. Each filament was uniquely labelled and resampled using the Bspline fit. Filaments within endpoints <10 nm from each other and that exhibited similar local orientations at the endpoints were reconnected as a single filament. The resampled actin points were exported with their XYZ coordinates, filament number and their position on the filament for further analysis.

Membrane segmentation was performed using Membrain-seg^108^. Regions of interest corresponding to the plasma membranes of adjacent cells were extracted in the Dragonfly software using the connected components analysis tool and cleaned up manually using the ROI painter. In most cases, both plasma membranes were separated and exported for further processing. For tomograms in which the plasma membranes are in close proximity to one another, the space between membranes was filled using the Fill Connected Pores tool in Dragonfly and the contour of this volume was generated using a custom Python script and processed with ColabSeg^109^ using outlier removal and DBSCAN clustering tools, yielding individual and mutually exclusive clusters for each plasma membrane. These were dilated by 5 nm and intersected with the original membrane segmentations generated in Membrain-seg. For plasma membranes along which endocytosis was observed (typically devoid of adjacent actin), the bud was replaced manually by a smooth patch of membrane connecting the area surrounding the endocytic site. Individual plasma membranes were imported into ColabSeg, and a radial basis function fit was applied to generate smooth, hole-free membrane surfaces for subsequent measurements.

Quantitative analysis of the cortex architecture was performed in Python using the coordinates of the equidistant sampled actin points and the membrane segmentation binary mask as input. Each actin point in the tomogram was assigned to one of the two cells captured in each tomogram based on its distance to the segmented plasma membranes. Subsequently, algorithms similar to those described by Jasnin et al.^110^ were used to analyse the local neighbourhood of each actin point in each cell. Actin bundles were detected based on the peak in the 2D histogram of the nearest neighbour distance and relative orientation. We identify actin bundles as two single actin points belonging to different filaments that are closer than 15 nm in distance with a relative filament orientation of less than 20° (Supplementary Fig. 5d and Supplementary Fig. 7c). The bundling fraction is given by bundled actin points over total actin points per cell. Branch site candidates were identified as locations at the end of a filament positioned within a distance of 15 nm from a second filament, where both filaments are positioned at an angle in the range between 50° and 90°, considering the 70° angle reported for Arp2/3 branching in literature^111,112^ (Supplementary Fig. 5c and Supplementary Fig. 7b). These candidates were further filtered by determining the approximate point of intersection of the two filaments by extrapolating the two-line segments and only including those that pass each other within a maximal distance of 3 nm. Additionally, all candidates where the closest points of two filaments were end points were removed because visual inspection showed those instances being typically false positives. A subset of detected branches was visually inspected in the tomograms by 3 experts giving a 16.6% false positive rate.

To determine the membrane-to-cortex distance, the distance to the closest membrane point was determined for each resampled actin point. The obtained distances were binned into 10-nm bins. The counts in each bin were normalized to the total counts per condition, to obtain the fraction of actin. Cells for which the nucleus was in close proximity to the plasma membrane were excluded from the analysis.

Figures and videos were generated using IMOD^105^ and Chimera X^113^.

### Filopodia quantification

Cells co-expressing CAmDia1 constructs of different sizes and the iMC-linker, CAezrin, or iMC-6FP were generated via lentiviral transduction and used in subsequent experiments as a mixed population. Cells were selected with antibiotics for 1 week for CAmDia1 expression. Upon dox-induction of the two constructs, double positive cells were sorted using the BD FACSAria™ Fusion cell sorter and directly seeded onto micropatterned glass-bottom dishes. Cells were fixed in PBS containing 4% PFA for 20 min at RT, washed twice with PBS, and permeabilized and blocked with 0.1% Triton-X-100 in PBS in the presence of 0.9% BSA for 15 min at RT. Cells were then stained with Phalloidin-AF647 (1:2,000) in blocking buffer at 4 °C until imaging.

Z-stacks of cells were acquired with 0.3 µm step size at a Nikon Ti-2^®^ CSU W1 SORA spinning disk with a 60x water immersion objective. Filopodia were quantified based on the presence of discrete mDia1-enriched spots located at the tips of cellular protrusions. Six representative 3D image volumes (dimensions approximately 70 x 800 x 800 voxels; voxel size 0.30 x 0.04 x 0.04 µm) were annotated using TrackMate^114^ (v7.13.0 doi:10.1038/s41592-022-01507-1, Fiji^115^), with the following parameters: estimated diameter = 0.4 µm, median filter enabled, sub-pixel localisation enabled, and manually optimized quality thresholds. Annotations were subsequently refined using Python (3.12.10) and Napari^116^. These curated datasets served as ground truth for training a deep learning–based spot detection model using Spotiflow^117^ (v0.5.8). The model was trained on the CAmDia1 single-channel 3D input, without the default down-sampling, and optimised for consistent performance across varying imaging conditions (key parameters: grid= 1, sigma= 3, pos-weight= 100). The trained model was applied to the full dataset for automated spot detection. Inference results were reviewed using a custom-built Napari interface Filospot v0.1.0 that enabled multi-channel visualisation and interactive filtering based on signal intensity across channels. Detected spots were manually cleaned to remove intracellular spot detection and spots overlaying with cortical signal, before coordinates were exported in CSV format and used to quantify the number of filopodia per cell.

### Numerical analysis, data visualisation and schematics

Numerical analyses and data visualisations were performed using the following software and packages: Microsoft Excel 16.78, RStudio 1.3.1093 and 2023.03.0 + 386 (tidyverse, ggplot2, ggbeeswarm, ggprism, ggbreak); Mathworks MATLAB 2019b; python 3.8.16; Jupyter 1.0.0; open3d 0.16.1; numpy 1.24.2; scipy 1.10.1; scikit-image 0.19.3; pandas 1.5.3; mrcfile 1.4.3; ipykernel 6.19.2; matplotlib 3.7.0; seaborn 0.12.2; tqdm 4.64.1; ChimeraX 1.4 (doi: 10.1002/pro.3943). Data Figures were composed in Adobe Illustrator^®^; presented schematics were generated in Adobe Illustrator^®^.

### Reporting summary

Further information on research design is available in the Nature Portfolio Reporting Summary linked to this article.

## Supplementary information

Supplementary Information

Description of Additional Supplementary Files

Supplementary Video 1

Reporting Summary

Transparent Peer Review file

## Source data

Source Data

## Data Availability

The dataset for the analysis of cortical actin and p-myosin in fixed cells, as well as actin and myosin−2 in live cells can be found together with the corresponding Fiji macro for immunofluorescence quantification in the Biostudies database (accession number S-BIAD2611). All raw frames, tilt series, metadata, tomograms and segmentations generated for this work are deposited in the Electron Microscopy Public Image Archive (EMPIAR)^118^ under accession code EMPIAR-13326. A representative tomogram associated with this entry is available in the Electron Microscopy Data Bank (EMDB)^119^ under entry EMD-56367. The experimental imaging data for the filopodia analysis, the used Spotiflow model with corresponding training data and example datasets are available in the BioStudies database (accession number S-BIAD2611). Raw numbers for plots presented in this paper as well as western blot images are available in the Source Data. All other data and unique reagents that support this study are available from the corresponding authors upon request. Source data are provided in this paper. Source data are provided with this paper.

## Code availability

The code used for cortex analysis from binary segmentations obtained during cryo-ET data processing is available on GitHub with the following link: https://github.com/MahamidLab/actin_cortex_analysis/tree/revision.

The Napari plugin for reviewing and manually correct spot detections used in the filopodia analysis is available at GitHub with the following link: https://github.com/diz-lab/filospot.
