# Supplementary material for: The membrane-to-cortex distance regulates mDia1 activity to control cortical mechanics
Source: Nat Commun. 2026 Sep 4;17:9501. doi: 10.1038/s41467-026-72845-3 (PMC13545285; doi:10.1038/s41467-026-72845-3)
Supplement: Supplementary file 1 [file 41467_2026_72845_MOESM1_ESM.pdf]

Supplementary information

The membrane-to-cortex distance regulates mDia1 activity to control cortical mechanics

Léanne Strauss[1,17], Sergio Lembo[1,9,17], Samuel F. Gérard[1,2], Marc Siggel[2,3,4], Dorothy Cheng[1,2], Martin Bergert[1], Sarah K. Foster[1], Joseph Vermeil[5,6,7,10,11], Mauricio Toro-Nahuelpan[1,2,11], Lena M. Fischer[1], Qin Yu[1,13], Ewa Sitarska[1,14,15], Chii Jou Chan[8,16], Jan Kosinski[2,3,4], Matthieu Piel[5,6], Olivia du Roure[7], Julien Heuvingh[7], Julia Mahamid[1,2], and Alba Diz-Muñoz[1,✉].

Affiliations

[1] Cell Biology and Biophysics Unit, European Molecular Biology Laboratory (EMBL); Heidelberg, Germany.
[2] Molecular Systems Biology Unit, EMBL; Heidelberg, Germany.
[3] EMBL Hamburg; Hamburg, Germany.
[4] Centre for Structural Systems Biology (CSSB); Hamburg, Germany.
[5] Institut Curie, PSL University, CNRS; Paris, France.
[6] Institut Pierre Gilles de Gennes, PSL University, CNRS; Paris, France.
[7] Physique et Mécanique des Milieux Hétérogènes (PMMH), CNRS, ESPCI Paris–Université PSL, Sorbonne Université, Université Paris Cité, F-75005 Paris, France
[8] Developmental Biology Unit, EMBL; Heidelberg, Germany.
[9] Present address: Institute of Science and Technology Austria (ISTA); Klosterneuburg, Austria.
[10] Present address: Laboratoire Jean Perrin, Institut de Biologie Paris-Seine, Sorbonne Université, 11 CNRS UMR 8237, Paris, France
[11] Present address: Institut Jacques Monod, CNRS UMR 7592
[12] Present address: Computational Innovation, Boehringer Ingelheim Pharma GmbH & Co. KG, Biberach, Germany
[13] Present address: The Francis Crick Institute, 1 Midland Road, NW1 1AT, London, UK
[14] Present address: Department of Cell Biology, Harvard Medical School, Boston, MA, USA
[15] Present address: Program in Cellular and Molecular Medicine, Boston Children's Hospital, Boston, MA, USA.
[16] Present address: Mechanobiology Institute, Department of Biological Sciences, National University of Singapore; Singapore.
[17] These authors contributed equally: Léanne Strauss, Sergio Lembo
Collaboration for joint PhD degree between EMBL and Heidelberg University, Faculty of Biosciences: Léanne Strauss, Sergio Lembo, Ewa Sitarska
✉ e-mail: diz@embl.de

Table of content

Supplementary Fig. 1: Biophysical characterization of the cell surface of NIH 3T3 fibroblasts and mESCs upon expression of different constructs, related to Fig. 1. 3

Supplementary Fig. 2: Quantification of cortical components in NIH 3T3 fibroblasts, related to Fig. 1. 5

Supplementary Fig. 3: Cryo-ET sample preparation, related to Fig. 2. 6

Supplementary Fig. 4: Additional exemplary -iMC tomograms to highlight the variability between datasets, related to Fig. 2. 7

Supplementary Fig. 5: Analysis of cortical architecture, related to Fig. 2 8

Supplementary Fig. 6: Additional exemplary +iMC tomograms to highlight the variability between datasets, related to Fig. 2. 9

Supplementary Fig. 7: Characterization of iMC-like-linkers of different lengths, related to Fig. 3. 10

Supplementary Fig. 8: Extended inhibitor panel and quantification of mDia1 levels, related to Fig. 4. 11

Supplementary Fig. 9: Detection of mDia1-nucleated filopodia, related to Fig. 4. 12

Supplementary Fig. 10: Predicted size of conventional membrane-to-cortex attachment proteins and exemplary activity of an unconventional one. 13

Supplementary Video 1: 13

Supplementary Table 1: Cortical tension of various iMC-linker-expressing clonal lines, related to Fig. 1. 14

Supplementary Table 3: Summary of tomography data. Related to Fig. 2 and Supplementary Fig. 3-6. 16

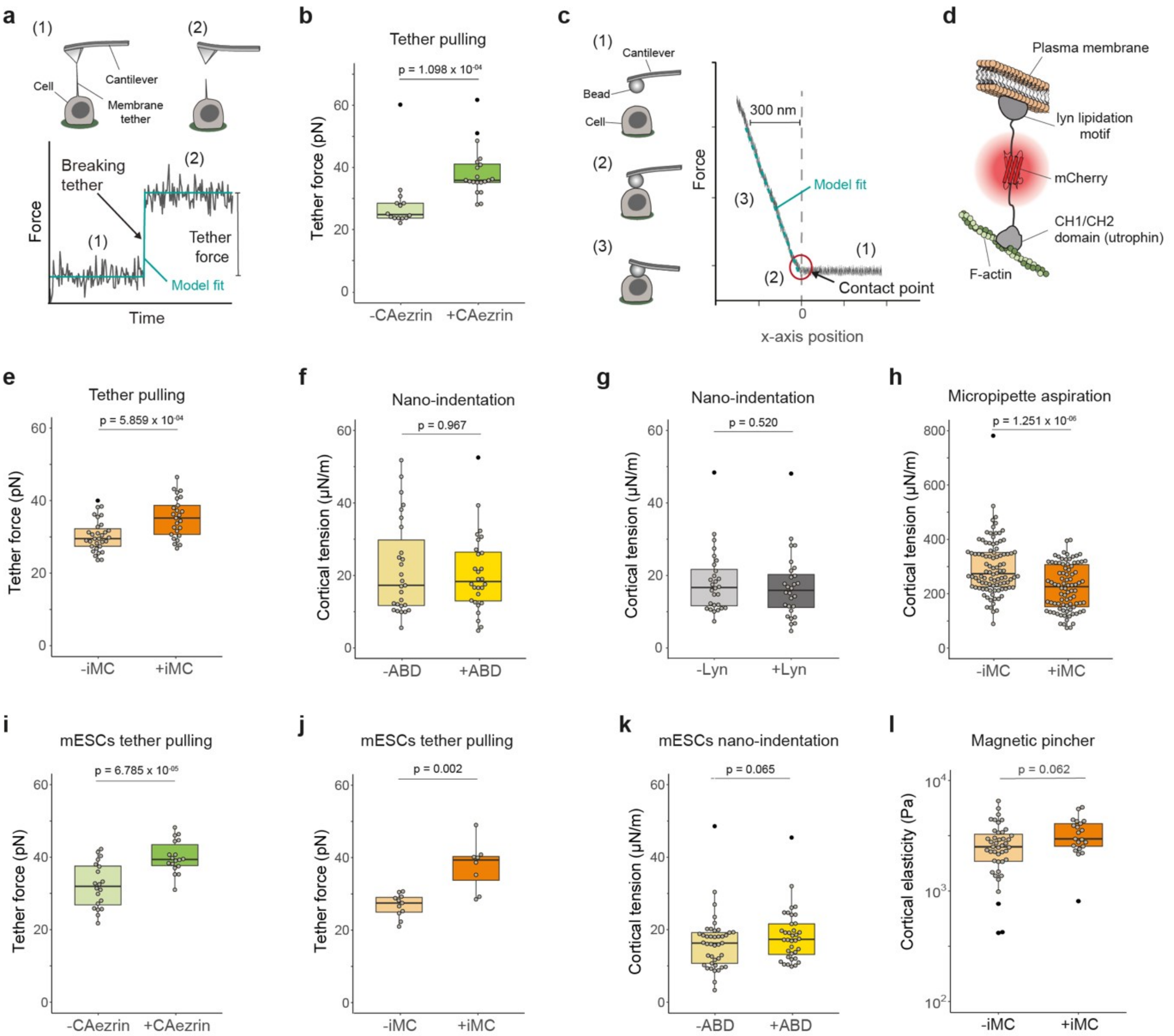

Supplementary Fig. 1: Biophysical characterization of the cell surface of NIH 3T3 fibroblasts and mESCs upon expression of different constructs, related to Fig. 1.

a, Schematic representation of static tether pulling with an atomic force spectrometer (top) and exemplary trace for tether pulling (bottom). Tether force is proportional to apparent membrane tension, and used here as a proxy for membrane-to-cortex attachment (see Methods).

b, Tether force in ± CAezrin NIH 3T3 fibroblasts; n_{Cells}: -CAezrin = 14, +CAezrin = 20; n_{Exp} = 3. **c,** Schematic representation of nano-indentation by atomic force spectrometry (left) with exemplary force-indentation curve for nano-indentation (right).

d, Schematic representation of the iMC-linker[30].

e, Tether force in ± iMC NIH 3T3 fibroblasts; n_{Cells}: -iMC = 31, +iMC = 25; n_{Exp} = 4.

f, Cortical tension of NIH 3T3 fibroblasts expressing the mCherry-tagged actin-binding domain of the iMC-linker (ABD); n_{Cells}: -ABD = 27, +ABD = 28; n_{Exp} = 3.

g, Cortical tension of NIH 3T3 fibroblasts expressing the plasma binding domain of the iMC-linker (lyn motif) tagged with mCherry (Lyn); n_{Cells}: -Lyn = 28, +Lyn = 28; n_{Exp} = 3.

h, Cortical tension measured with micropipette aspiration in ± iMC NIH 3T3 fibroblasts in suspension; n_{Cells}: -iMC = 96, +iMC = 89; n_{Exp} = 4.

i, **j,** Tether force upon expression of CAezrin **(i)** or the iMC-linker **(j)** in mESCs; n_{Cells}: -CAezrin = 20, +CAezrin = 18, -iMC = 11, +iMC = 8; n_{Exp}: CAezrin = 3, iMC = 2.

k, Cortical tension of mESCs expressing only the mCherry-tagged actin-binding domain of the iMClinker (ABD); n_{Cells}: -ABD = 41, +ABD = 36; n_{Exp} = 4.

l, Cortical elasticity in ± iMC NIH 3T3 fibroblasts; n_{Cells}: -iMC = 43, +iMC = 21; n_{Exp} = 4.

Box plots show the median (centre horizontal line), the lower and upper hinges correspond to the first and third quartiles (the 25th and 75th percentiles), whiskers extend from the hinge to the largest/smallest value, but no further than 1.5x interquartile range, outliers are marked by black dots, dots represent the mean of multiple measurements per single cell. n_{Cells} = number of analysed cells of each condition, n_{Exp} = number of independent experiments. Normality of data distribution was tested with the Shapiro-Wilk test. Two-tailed t-test was used for normally distributed data. Otherwise, a two-sided non-parametric Wilcox test was used.

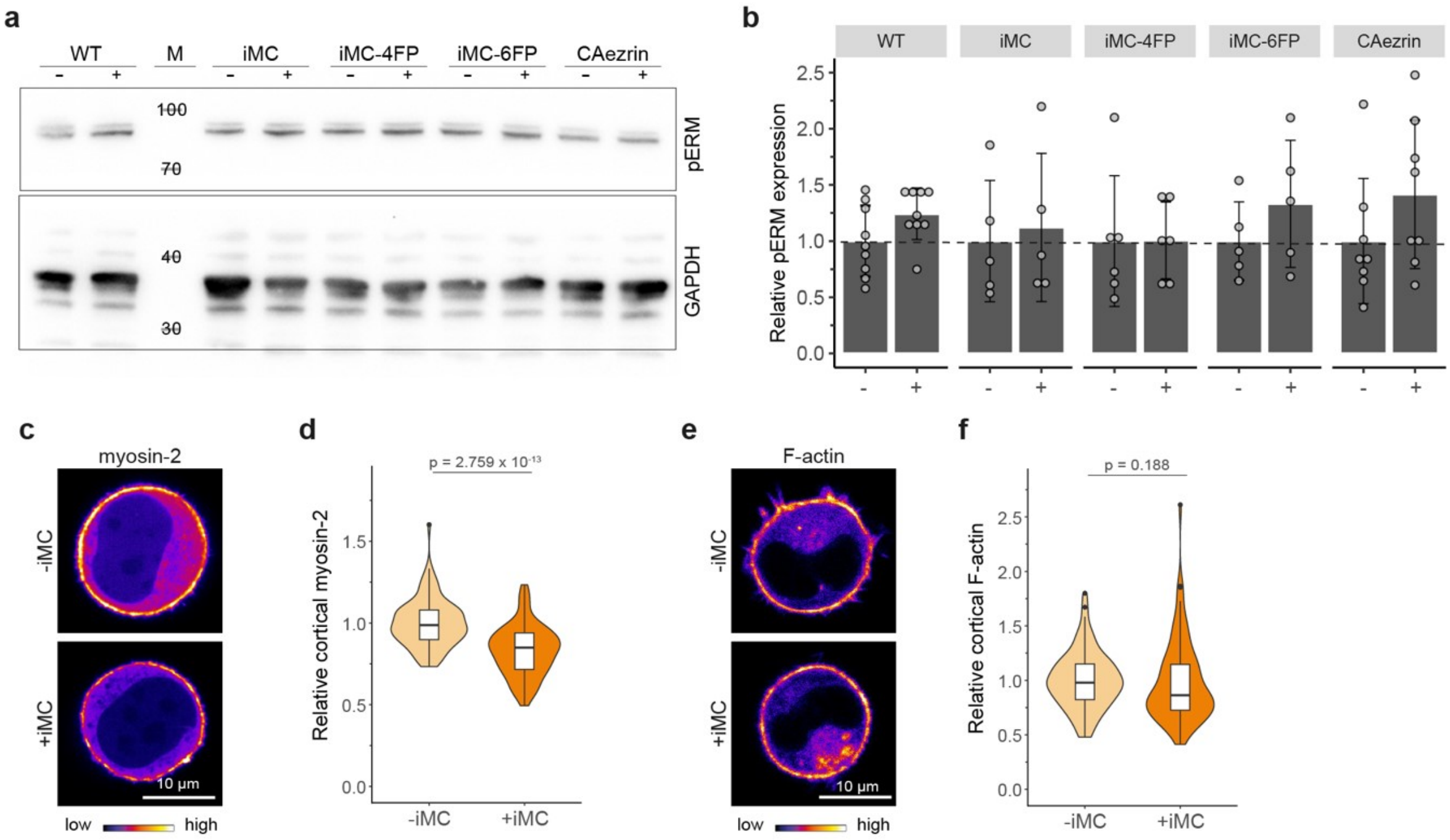

Supplementary Fig. 2: Quantification of cortical components in NIH 3T3 fibroblasts, related to Fig. 1.

a, Exemplary western blot showing the bulk expression of phosphorylated ERM proteins (pERM) upon expression of CAezrin or iMC-like-linker constructs. Addition of doxycycline (+) induces expression of the indicated construct, except in wild type (WT) cells, which contain no construct. M = molecular weight marker in kDa.
b, Quantification of pERM expression upon expression of the indicated construct (+) based on the signal detected by western blot; the signal for each lane was normalized to the respective GAPDH signal, protein expression is shown relative to the respective non-induced control (-). Bars show the mean of different replicates and error bars the standard deviation, dots correspond to individual experiments; n_{Exp}: WT = 9, iMC = 5, iMC-4FP = 6, iMC-6FP = 5, CAezrin = 8.
c, Representative confocal XY plane of cortical myosin-2 in live ±iMC NIH 3T3 fibroblasts labelled with a fluorescently-tagged myosin light chain[36].
d, Quantification of cortical myosin-2 amount, displayed is the mean fluorescent intensity normalized to mean of -iMC cells; n_{Cells}: -iMC = 100, +iMC = 108; n_{Exp} = 3.
e, Representative confocal XY plane of cortical F-actin in live ± iMC NIH 3T3 fibroblasts stained with SPY650-FastAct^{TM}.
f, Quantification of cortical F-actin amount, displayed is the mean fluorescence intensity normalized to mean of -iMC cells; n_{Cells}: -iMC = 75, +iMC = 79; n_{Exp} = 4.
The coloured area in the violin plot reflects the density distribution of data points. Box plots show the median (centre horizontal line), the lower and upper hinges correspond to the first and third quartiles (the 25th and 75th percentiles), whiskers extend from the hinge to the largest/smallest value, but no further than 1.5x interquartile range, outliers are marked by black dots. n_{Cells} = number of analysed cells of each condition, n_{Exp} = number of independent experiments. Normality of data distribution was tested with the Shapiro-Wilk test. Statistical significance was tested using a two-sided non-parametric Wilcox test.

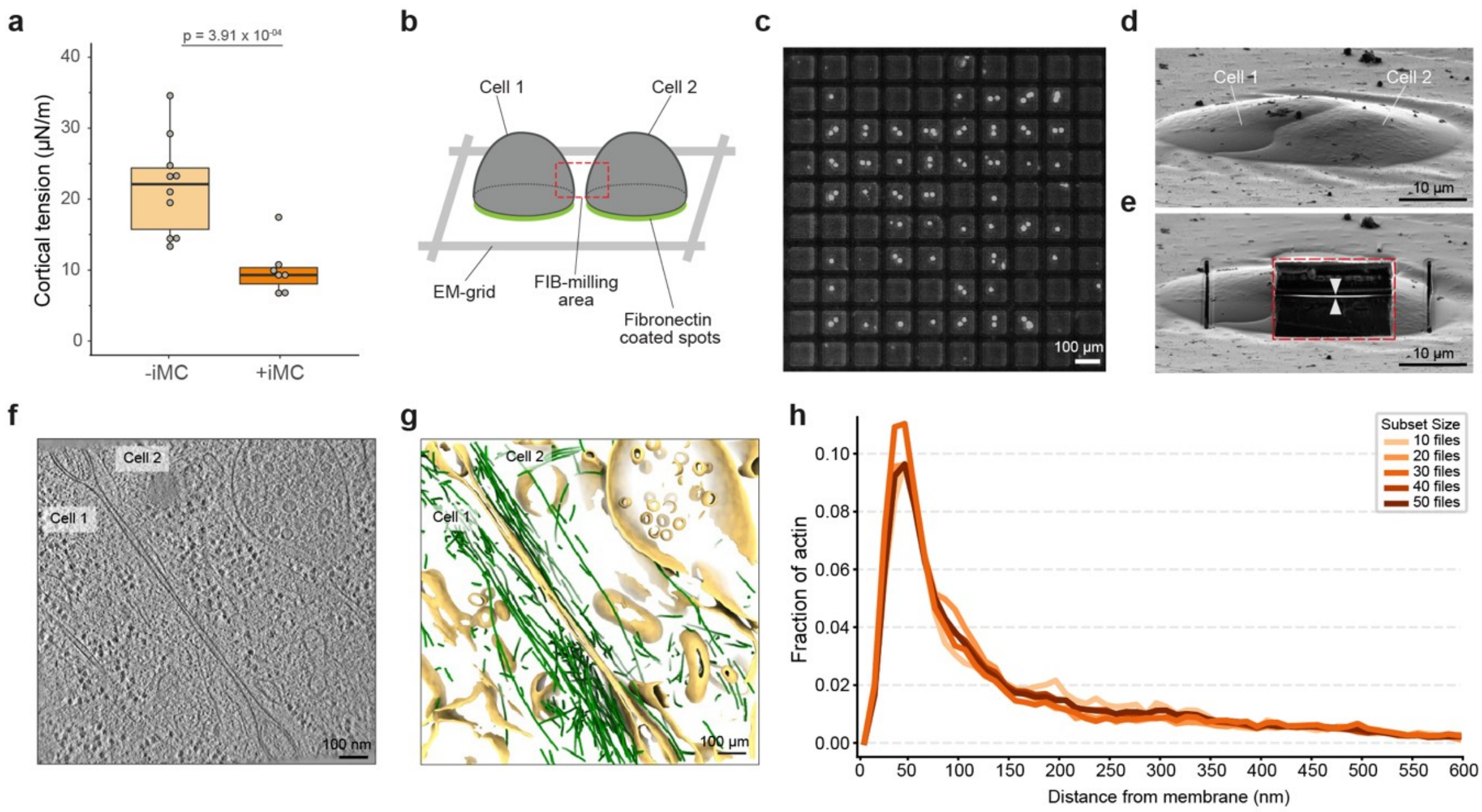

Supplementary Fig. 3: Cryo-ET sample preparation, related to Fig. 2.

a, Cortical tension measured by nano-indentation of cells on EM-grids. Dots represent the mean of multiple measurements per single cell; n_{Cells}: -iMC = 10, +iMC = 7; n_{Exp} = 1.
b, Schematic representation of a cell pair seeded on fibronectin coated spots within an EM-grid square. The pattern is designed to accommodate two NIH 3T3 fibroblasts in close proximity to allow cryo-FIB lamella generation through two neighbouring cells, position is marked by the dashed red square.
c, Representative phase-contrast image of unlabelled NIH 3T3 fibroblasts on micropatterned EM-grids.
d, e, FIB-SEM image of a representative cell pair seeded on a micropatterned EM-grid square after plunge-freezing (**d**). The grids underwent 3 s blotting before plunge-freezing, which leads to mild flattening of the cell. Generation of a ~200-nm-thick lamella (**e**, white arrows) by FIB ablation of material at the bottom and top of the cell (dashed red square). The two thin trenches on the sides are designed to relieve strain and mitigate bending of the thin lamella.
f, Representative tomogram slice.
g, Segmentation of the tomogram shown in **e**, actin filaments in green, membranes in yellow.
h, Spatial distribution of actin in control cells. Lines display the pooled distribution of actin filaments from a random subset of cellular regions ($n_{Cell\ regions}$ =10 to 50) as a function of distance from the membrane.
Box plot shows the median (centre horizontal line), the lower and upper hinges correspond to the first and third quartiles (the 25th and 75th percentiles), whiskers extend from the hinge to the largest/smallest value, but no further than 1.5x interquartile range, outliers are marked by black dots, dots represent the mean of multiple measurements per single cell. $n_{Cell\ regions}$ = number of analysed cellular regions (1-2 in each tomogram), n_{Cells} = number of analysed cells of each condition, n_{Exp} = number of independent experiments. Normality of data distribution was tested by Shapiro-Wilk test. Statistical significance was calculated using a two-tailed t-test.

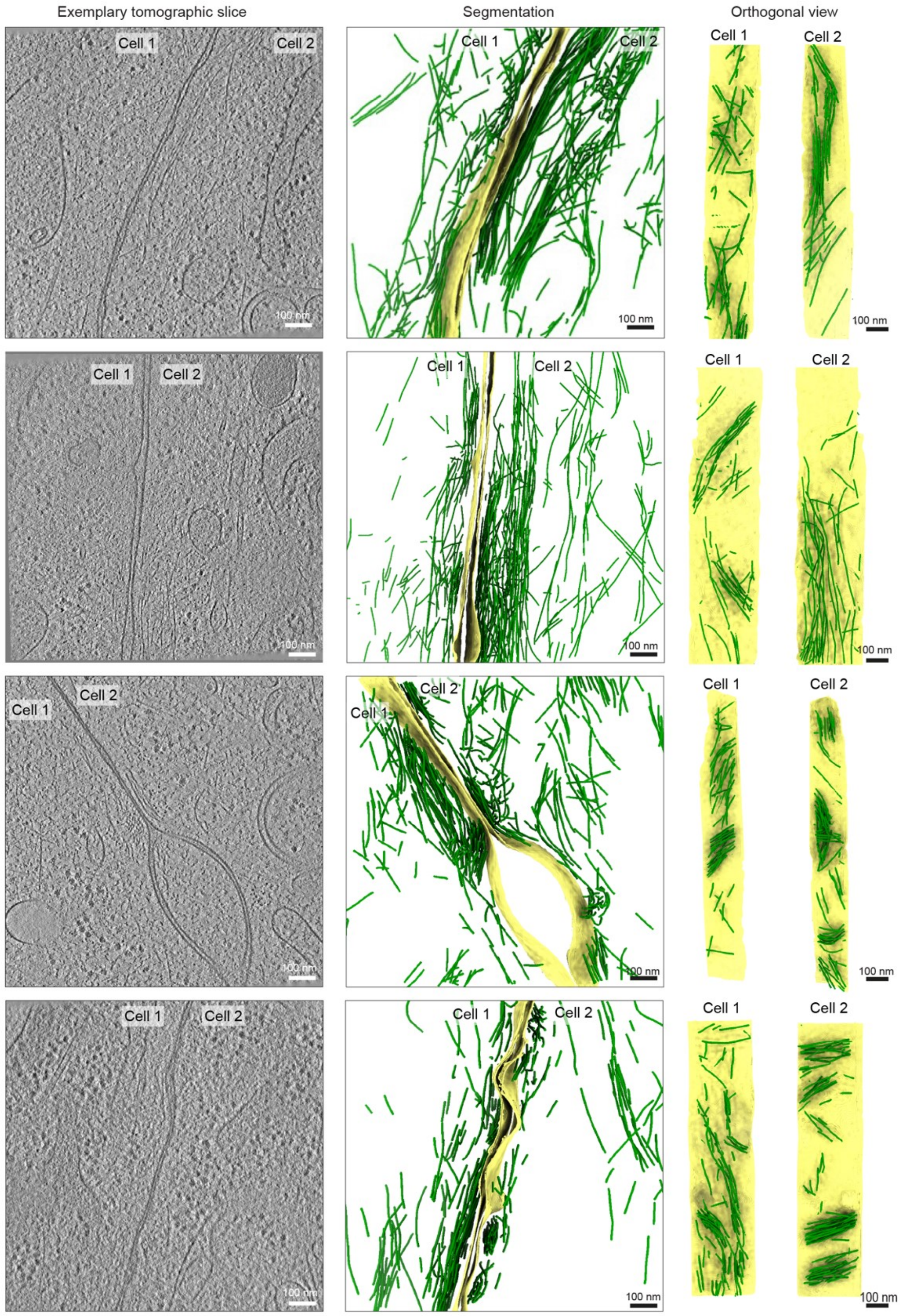

Supplementary Fig. 4: Additional exemplary -iMC tomograms to highlight the variability between datasets, related to Fig. 2.

Additional exemplary tomographic slices through two adjacent control cells. Corresponding segmentation of actin filaments (green) and plasma membrane (yellow) of the full tomogram and orthogonal views showing actin filaments within 50 nm of the membrane.

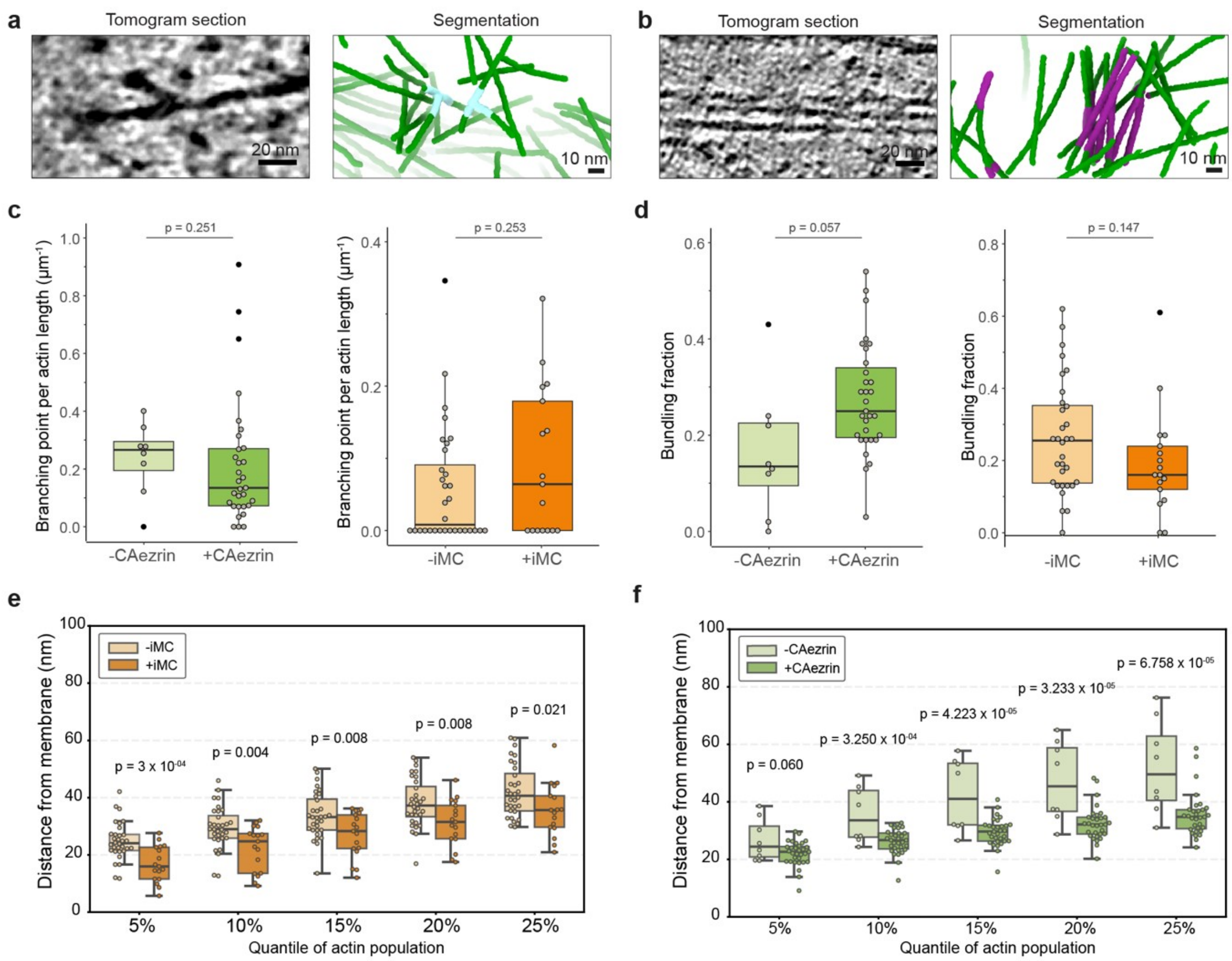

Supplementary Fig. 5: Analysis of cortical architecture, related to Fig. 2

a, Example of an F-actin branch point and segmentation of branched actin in a different tomogram; F-actin in green and detected branch point in cyan.

b, Example of an F-actin bundle and segmentation of bundled actin in a different tomogram; F-actin in green and detected bundles in purple.

c, d, Number of F-actin branches per actin length (**c**), and bundling fraction (**d**) calculated for cells expressing the iMC-linker or CAezrin in comparison to respective control cells; $n_{Cell\ region}$: -CAezrin = 8, +CAezrin = 31, -iMC = 32, +iMC = 17, n_{Cells}: -CAezrin = 7, +CAezrin = 15, -iMC = 15, +iMC = 9.

e, f, Quantile analysis of actin point distances from the membrane for cells expressing the iMC-linker (**e**) or CAezrin (**f**) compared to the respective control. For each cellular region, the 5th, 10th, 15th, 20th and 25th percentiles (quantiles) of the actin distance distribution were calculated. $n_{Cell\ region}$: -CAezrin = 8, +CAezrin = 31, -iMC = 32, +iMC = 17, n_{Cells}: -CAezrin = 7, +CAezrin = 15, -iMC = 15, +iMC = 9.

Box plots show the median (centre horizontal line), the lower and upper hinges correspond to the first and third quartiles (the 25th and 75th percentiles), whiskers extend from the hinge to the largest/smallest value, but no further than 1.5x interquartile range, outliers are marked by black dots, dots represent individual cellular regions. $n_{Cell\ region}$: number of analysed cellular regions (1-2 in each tomogram). We obtained 1-4 tomograms per lamella, due to the high variability between tomograms (Supplementary Fig. 4 and 6), cellular regions in tomograms from a single lamella were considered independent data points. n_{Cells} = number of analysed cells. For **c** and **d**, normality of data distribution was tested by Shapiro-Wilk test. Two-tailed t-test was used for normally distributed data. Otherwise, a two-sided non-parametric Wilcox test was used. For **e** and **f**, statistical significance was determined using a linear mixed-effects model for each quantile, treating the conditions as a fixed effect and cellular regions as a random effect.

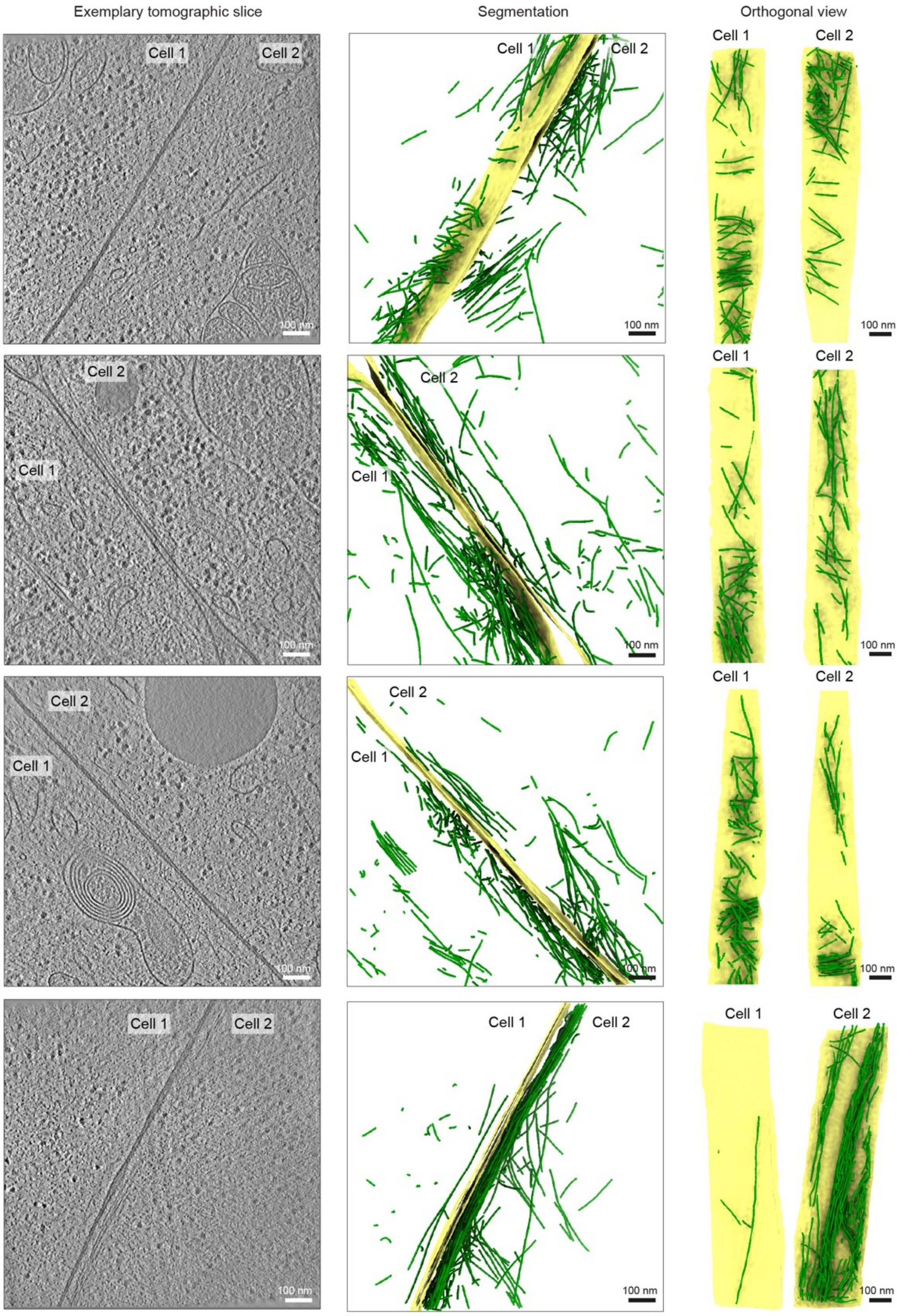

Supplementary Fig. 6: Additional exemplary +iMC tomograms to highlight the variability between datasets, related to Fig. 2.

Additional exemplary tomographic slices through two adjacent iMC-linker-expressing cells. Corresponding segmentation of actin filaments (green) and plasma membrane (yellow) of the full tomogram and orthogonal views showing actin filaments within 50 nm of the membrane.

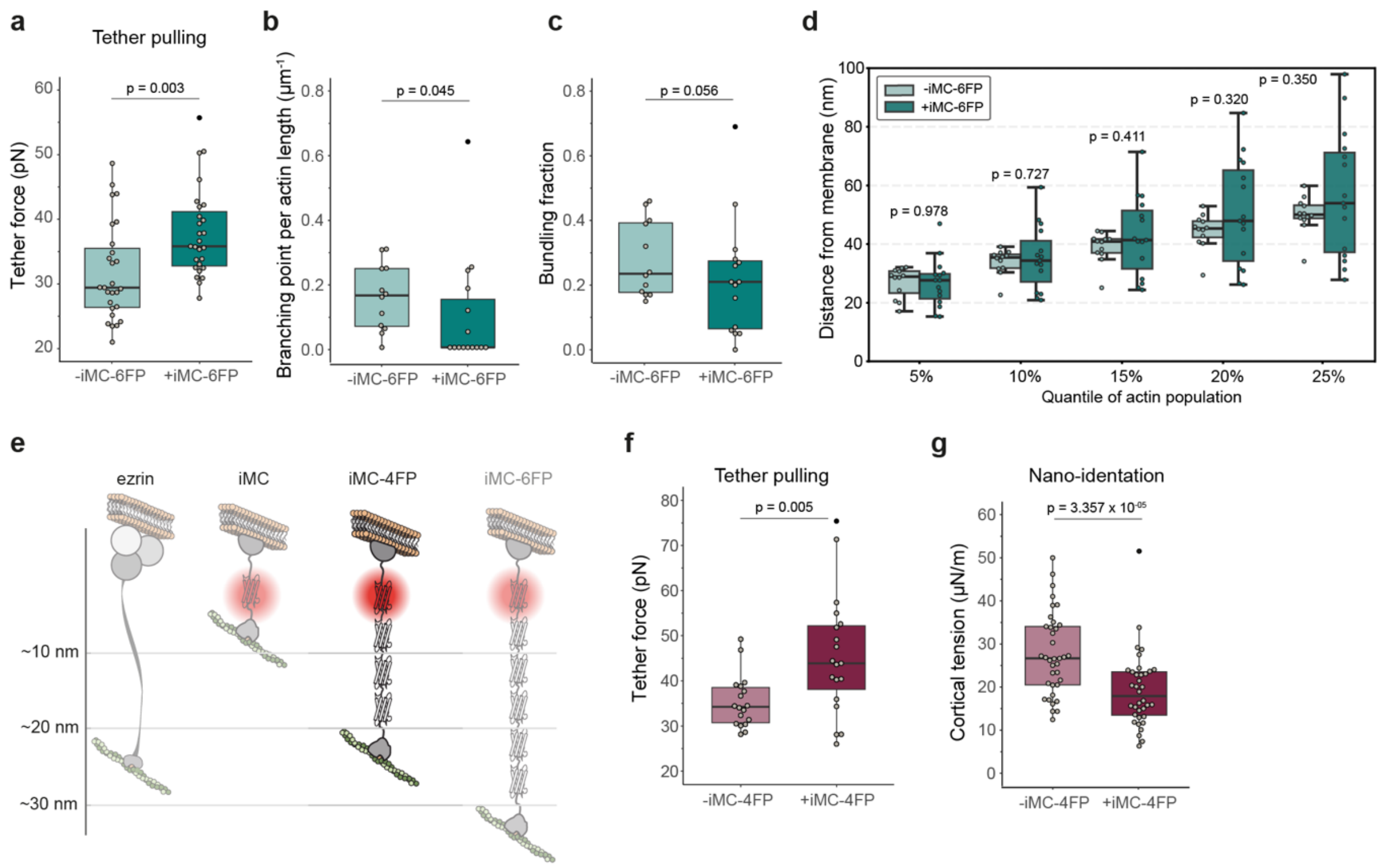

Supplementary Fig. 7: Characterization of iMC-like-linkers of different lengths, related to Fig. 3.

a, Tether force for ±iMC-6FP-linker NIH 3T3 fibroblasts; dots represent the mean of multiple measurements per single cell; n_{Cells}: -iMC-6FP= 27, +iMC-6FP = 27; n_{Exp} = 3.

b, **c,** Branches per unit actin length (**b**), and bundling fraction (**c**) for ± iMC-6FP-linker cells, dots represent individual cellular regions.; $n_{Cell\ region}$: -iMC-6FP = 12, +iMC-6FP = 15, n_{Cells}: -iMC-6PF = 6, +iMC-6FP = 10.

d, Quantile analysis of actin point distances from the membrane for cells expressing the iMC-6FP-linker compared to control cells. For each cellular region, the 5th, 10th, 15th, 20th and 25th percentiles (quantiles) of the actin distance distribution were calculated, dots represent individual cellular regions. $n_{Cell\ region}$: -iMC-6FP = 12, +iMC-6FP = 15, n_{Cells}: -iMC-6PF = 6, +iMC-6FP = 10. Statistical significance was determined using a linear mixed-effects model for each quantile, treating the conditions as a fixed effect and cellular regions as a random effect.

e, Schematic representation of all iMC-like-linker constructs showing their approximated length at full extension in comparison to CAezrin.

f, Tether force for ± iMC-4FP-linker NIH 3T3 fibroblasts, dots represent the mean of multiple measurements per single cell; n_{Cells}: -iMC-4FP = 18, +iMC-4FP = 19; n_{Exp} = 2.

g, Cortical tension of ±iMC-4FP-linker NIH 3T3 fibroblasts, dots represent the mean of multiple measurements per single cell; n_{Cells}: -iMC-4FP = 37, +iMC-4FP = 37; n_{Exp} = 4.

Box plots show the median (centre horizontal line), the lower and upper hinges correspond to the first and third quartiles (the 25th and 75th percentiles), whiskers extend from the hinge to the largest/smallest value, but no further than 1.5x interquartile range, outliers are shown by black dots. $n_{Cell\ regions}$ = number of analysed cellular regions (1-2 in each tomogram), we obtained 1-4 tomograms per lamella, due to the high variability between tomograms, cellular regions in tomograms from a single lamella were considered independent data points (Supplementary Fig. 4 and 6), n_{Cells} = number of analysed cells, n_{Exp}= number of independent experiments. If not stated otherwise, normality of data distribution was tested by Shapiro-Wilk test. Two-tailed t-test was used for normally distributed data. Otherwise, a nonparametric Wilcox test was used.

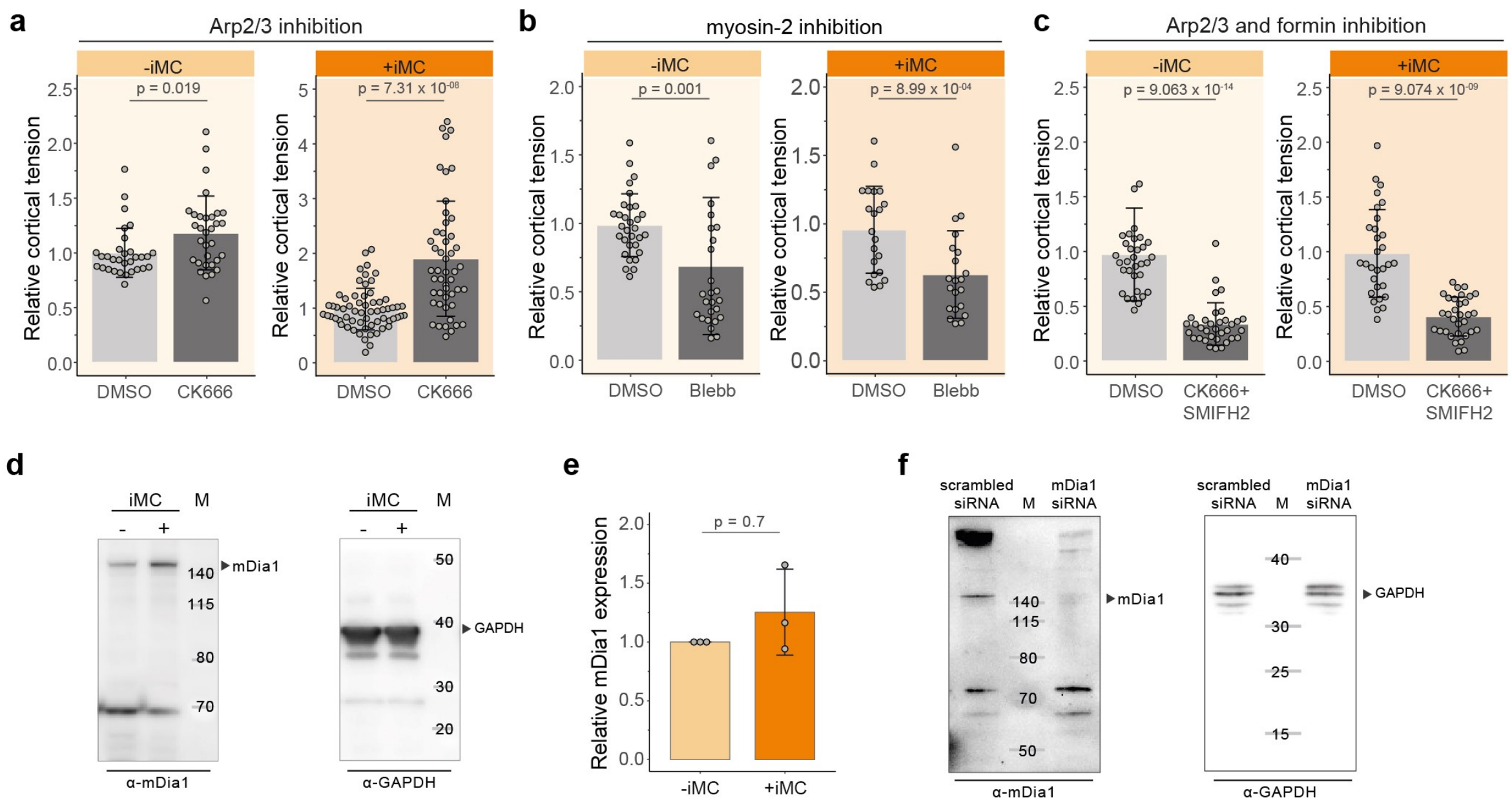

Supplementary Fig. 8: Extended inhibitor panel and quantification of mDia1 levels, related to Fig. 4.

a, Relative cortical tension of ± iMC NIH 3T3 fibroblasts upon treatment with the Arp2/3 complex inhibitor CK666, normalized per replicate to the mean cortical tension of DMSO treated cells; n_{Cells}: -iMC+DMSO = 31, -iMC+CK666 = 32, +iMC+DMSO = 62, +iMC+CK666 = 49; n_{Exp}: -iMC = 3, +iMC = 6.
b, Relative cortical tension of ± iMC NIH 3T3 fibroblasts upon treatment with the myosin inhibitor Blebbistatin (Blebb), normalized per replicate to the mean cortical tension of DMSO treated cells; n_{Cells}: -iMC+DMSO = 31, -iMC+Blebb = 26, +iMC+DMSO = 21, +iMC+Blebb = 21; n_{Exp}: -iMC = 3, +iMC = 2.
c, Relative cortical tension of ± iMC NIH 3T3 fibroblasts upon co-treatment with the Arp2/3 complex inhibitor CK666 and the pan-formin inhibitor SMIFH2, normalized per replicate to the mean cortical tension of DMSO treated cells; n_{Cells}: -iMC+DMSO = 33, -iMC+CK666+SMIFH2 = 34, +iMC+DMSO = 30, +iMC+CK666+SMIFH2 = 32; n_{Exp}: -iMC = 3, +iMC = 3.
d, Representative uncut western blot, the upper half of the membrane was stained with α-mDia1 antibody (left) the lower part with α-GAPDH antibody (right). For quantification of protein expression levels, the signal indicated by arrowheads was used. M = molecular weight marker in kDa.
e, Quantification of mDia1 protein upon induction of the iMC-linker based on the signal detected by western blot; the signal for each lane was normalized to the respective GAPDH signal, protein expression is shown relative to control cells (-iMC); dots show independent experiments, n_{Exp} = 3.
f, Representative uncut western blot upon siRNA knockdown of mDia1, the upper half of the membrane was stained with α-mDia1 antibody (left) the lower part with α-GAPDH antibody (right). For quantification of protein expression levels (see Fig. 4e), the signal indicated by arrowheads was used. M = molecular weight marker in kDa.
In all plots, bars show the mean and error bars the standard deviation. If not stated otherwise, dots represent the mean of multiple measurements per single cell. n_{Cells} = number of analysed cells of each condition, n_{Exp} = number of independent experiments. Normality of data distribution was tested by Shapiro-Wilk test. Two-tailed t-test was used for normally distributed data. Otherwise, a two-sided nonparametric Wilcox test was used.

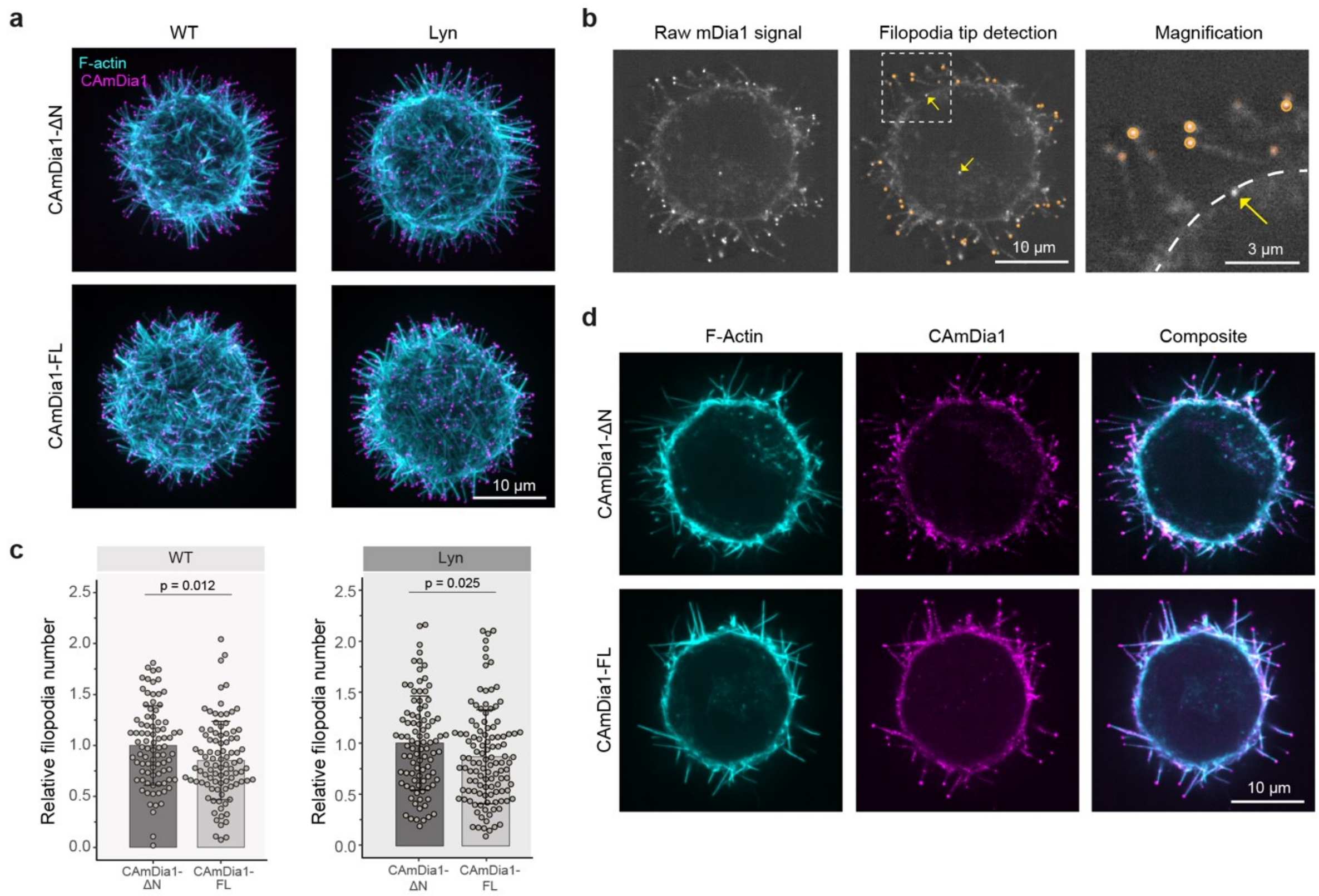

Supplementary Fig. 9: Detection of mDia1-nucleated filopodia, related to Fig. 4.

a, Representative maximum intensity projections of fixed control cells: cells expressing CAmDia1 constructs only (wild type, WT) and cells co-expressing CAmDia1 constructs and an mCherry tagged lyn motif (Lyn).

b, Number of filopodia per cell determined by automated spot detection (for more details see Methods), values were normalized to the mean filopodia number of cells expressing CAmDia1-ΔN; n_{Cells}: control+CAmDia1-ΔN = 86, control+CAmDia1-FL = 93, +Lyn+CAmDia1-ΔN = 93, +Lyn+CAmDia1-FL = 115; n_{Exp}: WT = 3, +Lyn = 4.

c, Automated spot detection with Spotiflow[117], example z-slice of raw CAmDia1 constructs signal with detected spots highlighted with orange circles. Smaller circles indicate spot detection in adjacent slices, intracellular or cortically localised spots (marked by yellow arrows) were removed; magnification of dashed square is shown; cortex is indicated by a dashed semi-circle in the magnification.

d, Representative images of cells co-expressing the iMC-linker and the indicated CAmDia1 construct. Bar plots show the mean and error bars the standard deviation of normalized values, dots represent individual cells. n_{Cells} = number of analysed cells of each condition, n_{Exp} = number of independent experiments. Normality of data distribution was tested by Shapiro-Wilk test. Two-tailed t-test was used for normally distributed data. Otherwise, a non-parametric Wilcox test was used.

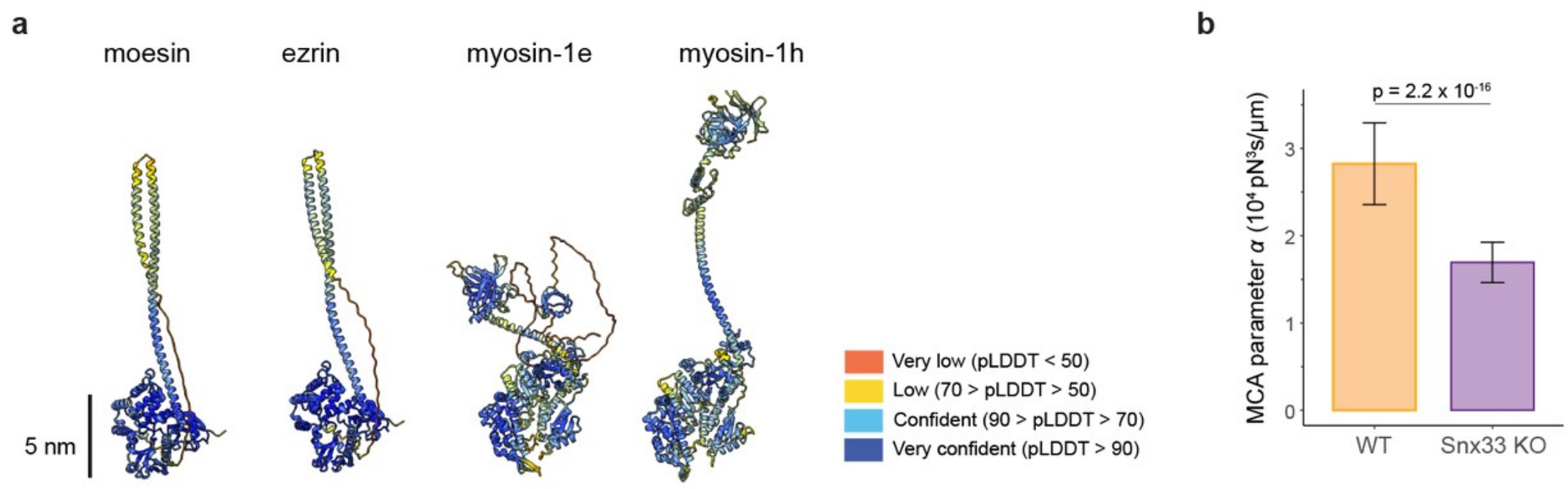

Supplementary Fig. 10: Predicted size of conventional membrane-to-cortex attachment proteins and exemplary activity of an unconventional one.

a, AlphaFold2[73] predictions of canonical membrane-to-cortex attachment linker proteins, highlighting their diversity in size; as a measure of prediction confidence, the predicted local distance difference test (pLDDT) is colour-coded.
b, Membrane-to-cortex attachment of neutrophil-like HL-60 cells with (wild type, WT) and without (knockout, KO) the BAR-domain protein Snx33 measured by dynamic tether pulling, MCA = membrane-to-cortex attachment; bars show mean, error bars display standard deviation; n_{Cells}: WT = 12, Snx33 KO = 12; n_{Exp} = 3. n_{Cells} = number of analysed cells of each condition, n_{Exp} = number of independent experiments. Normality of data distribution was tested by Shapiro-Wilk test. Statistical significance was tested using a two-tailed non-parametric Wilcox test.

Supplementary Video 1:

Tomogram of a region transecting two adjacent NIH 3T3 fibroblasts, showing two cell membranes and two cortices. This tomogram is shown in Fig. 2a as a single representative slice. Segmentation of actin (green) and plasma membrane (yellow) is overlayed on the tomogram. Scale bar is 100 nm.

Supplementary Table 1: Cortical tension of various iMC-linker-expressing clonal lines, related to Fig. 1.

Data from Fig. 1d displaying normalised cortical tension and iMC-linker density for various clonal lines. Given are the number of analysed cells of each condition (n_{Cells}) and number of independent experiments (n_{Exp}) for each clone.

Clone	Relative cortical tension	iMC-linker density (a.u.)	n_{Cells} -iMC	n_{Cells} +iMC	n_{Exp}
iMC-1	0.275	13376.3	30	31	3
iMC-2	0.370	13117.0	34	34	3
iMC-3	0.379	5551.0	52	51	5
iMC-4	0.651	3842.7	18	18	2
iMC-5	0.862	3800.7	24	27	3

Supplementary Table 2: Constructs used in this study.

Construct name	Description	Cell line
CAezrin	constitutively active human ezrin (T567D)-mCherry[23]	Clonal cell line in NIH 3T3 and mESC background
iMC-linker	lyn lipidation motif-mCherry-CH1/CH2 (human utrophin)[30]	Clonal cell lines in NIH 3T3 and mESC background
Lyn	lyn lipidation motif tagged with mCherry[32]	Clonal cell line in NIH 3T3 background
ABD	mCherry-CH1/CH2 domain (human utrophin)[31]	Clonal cell line in NIH 3T3 and mESC background
iMC-6FP-linker	lyn lipidation motif-mCherry-dark mCherry(x5)-CH1/CH2 (human utrophin) – generated for this study	Clonal cell line in NIH 3T3 background
iMC-4FP-linker	lyn lipidation motif-mCherry-dark mCherry(x3)-CH1/CH2 (human utrophin) – generated for this study	Clonal cell line in NIH 3T3 background
CAmDia1-ΔN	N-terminal-truncated mDia1 construct tagged with eGFP, generated via Gibson assembly from CAmDia1-FL. A C-terminally lyn lipidation motif was added for plasma membrane targeting – generated for this study	Cell line in +iMC, +CAezrin, +iMC6FP background
CAmDia1-FL	mDia1 full length sequence tagged by eGFP; constitutively active though two point mutations in its DAD domain[63]	Cell line in +iMC, +CAezrin, +iMC6FP background

Supplementary Table 3: Summary of tomography data. Related to Fig. 2 and Supplementary Fig. 3-6.

Condition	-iMC	+iMC	-CAezrin	+CAezrin	-iMC-6FP	+iMC-6FP
Number of grids	3	3	2	3	1	2
Number of lamellae imaged	7	5	5	11	3	6
Number of tomograms	17	9	6	20	8	10
Number of cellular regions	32	17	8	31	12	15
Defocus range	-2 to -4 µm (VPP)	-2 to -4 µm (VPP)	-2 to -4 µm (VPP)	-2 to -4 µm (VPP)	-6 to -8 µm (defocus)	-6 to -8 µm (defocus)
Number of cells analysed	15	9	7	15	6	10
Number of traced actin filaments	3428	2084	919	5049	2146	2095
Number of actin points	148287	77424	23933	161041	72052	67640
