# Supplementary material for: The membrane-to-cortex distance regulates mDia1 activity to control cortical mechanics
Source: Nat Commun. 2026 Sep 4;17:9501. doi: 10.1038/s41467-026-72845-3 (PMC13545285; doi:10.1038/s41467-026-72845-3)
Supplement: Supplementary file 2 [file 41467_2026_72845_MOESM2_ESM.pdf]

Title: Supplementary Video 1

Description: Tomogram of a region transecting two adjacent NIH 3T3 fibroblasts, showing two cell membranes and two cortices. This tomogram is shown in Fig. 2a as a single representative slice. Segmentation of actin (green) and plasma membrane (yellow) is overlayed on the tomogram. Scale bar is 100 nm.
