# Supplementary material for: The membrane-to-cortex distance regulates mDia1 activity to control cortical mechanics
Source: Nat Commun. 2026 Sep 4;17:9501. doi: 10.1038/s41467-026-72845-3 (PMC13545285; doi:10.1038/s41467-026-72845-3)
Supplement: Supplementary file 3 [file 41467_2026_72845_MOESM4_ESM.pdf]

nature portfolio

Corresponding author(s): Dr. Alba Diz-Muñoz

Last updated by author(s): 09.04.2026

Reporting Summary

Nature Portfolio wishes to improve the reproducibility of the work that we publish. This form provides structure for consistency and transparency in reporting. For further information on Nature Portfolio policies, see our Editorial Policies and the Editorial Policy Checklist.

Statistics

For all statistical analyses, confirm that the following items are present in the figure legend, table legend, main text, or Methods section.

n/a	Confirmed	
☐	☒	The exact sample size (n) for each experimental group/condition, given as a discrete number and unit of measurement
☐	☒	A statement on whether measurements were taken from distinct samples or whether the same sample was measured repeatedly
☐	☒	The statistical test(s) used AND whether they are one- or two-sided<br>*Only common tests should be described solely by name; describe more complex techniques in the Methods section.*
☒	☐	A description of all covariates tested
☐	☒	A description of any assumptions or corrections, such as tests of normality and adjustment for multiple comparisons
☐	☒	A full description of the statistical parameters including central tendency (e.g. means) or other basic estimates (e.g. regression coefficient) AND variation (e.g. standard deviation) or associated estimates of uncertainty (e.g. confidence intervals)
☐	☒	For null hypothesis testing, the test statistic (e.g. F, t, r) with confidence intervals, effect sizes, degrees of freedom and P value noted<br>*Give P values as exact values whenever suitable.*
☒	☐	For Bayesian analysis, information on the choice of priors and Markov chain Monte Carlo settings
☒	☐	For hierarchical and complex designs, identification of the appropriate level for tests and full reporting of outcomes
☒	☐	Estimates of effect sizes (e.g. Cohen's d, Pearson's r), indicating how they were calculated

Our web collection on statistics for biologists contains articles on many of the points above.

Software and code

Policy information about availability of computer code

Data collection	AFM: JPK CellHesion200 (v.6)<br><br>Immunofluorescence imaging: Leica Application Suite X 4.8.1.29271, NIS Elements AR 5.30.07<br><br>Magnetic Pincher and Micropipette aspiration: custom-made Labview (National Instruments) software<br><br>Micropatterning on glass and EM grids: Nikon Eclipse Ti and Alveole Primo micropatterning system operated with Leonardo 4.16 through µManager 1.4.22 (doi: 10.14440/jbm.2014.36)<br><br>Cryo-FIB milling: Thermo Fisher Scientific Aquilos operated with SerialFIB 1.0 (github.com/sklumpe/SerialFIB)<br><br>Cryo-electron tomography: Thermo Fisher Scientific Krios operated with SerialEM 4.1.0beta (doi: 10.1016/j.jsb.2005.07.007), TFS Krios +SerialEM OK, K2 Summit direct electron detector (Gatan), Volta phase plate (ThermoFisher Scientific)<br>Volta phase plate (ThermoFisher Scientific)
Data analysis	AFM data: JPK SPM Desktop and Processing (version 6.4.39)<br><br>Image analysis: ImageJ2, version 2.9.0/1.54b, MorpholibJ plugin<br><br>Cryo-electron tomography: Warp 1.0.9 and 1.0.7 (doi: 10.1038/s41592-019-0580-y); IMOD version 4.9 (doi: 10.1006/jsbi.1996.0013); DeePiCt

1.0.0 (for spectrum filtering (github.com/ZauggGroup/DeePiCt)); Thermo Fisher Scientific Amira 2022.1; Colabseg; MemBrain-seg; Dragonfly software version 2022.2; The cryo-ET analysis code is available on GitHub with the following link: https://github.com/MahamidLab/actin_cortex_analysis/tree/revision.

Numerical data analysis and visualisation: Microsoft Excel 16.78, RStudio 1.3.1093 and 2023.03.0+386 (tidyverse, ggplot2, ggbeeswarm, ggprism, ggbreak); Mathworks MATLAB 2019b; python 3.8.16; Jupyter 1.0.0; open3d 0.16.1; numpy 1.24.2; scipy 1.10.1; scikit-image 0.19.3; pandas 1.5.3; mrcfile 1.4.3; ipykernel 6.19.2; matplotlib 3.7.0; seaborn 0.12.2; tqdm 4.64.1; ChimeraX 1.4 (doi: 10.1002/pro.3943)

Filopodia analysis: Python 3.12.10, Napari, TrackMate v7.13.0 doi:10.1038/s41592-022-01507-1, Spotiflow v0.5.8, Napari-Spotiflow v0.4.1 Filospot v0.1.0; The Napari plugin for reviewing and manually correct spot detections is available at GitHub (https://github.com/diz-lab/filospot).

For manuscripts utilizing custom algorithms or software that are central to the research but not yet described in published literature, software must be made available to editors and reviewers. We strongly encourage code deposition in a community repository (e.g. GitHub). See the Nature Portfolio guidelines for submitting code & software for further information.

Data

Policy information about availability of data

All manuscripts must include a data availability statement. This statement should provide the following information, where applicable:

- Accession codes, unique identifiers, or web links for publicly available datasets
- A description of any restrictions on data availability
- For clinical datasets or third party data, please ensure that the statement adheres to our policy

Datasets for the analysis of cortical actin and p-myosin in fixed cells, as well as actin and myosin in live cells can be found together with the corresponding Fiji macro for immunofluorescence quantification in the Biostudies database (accession number S-BIAD2611).

The presented cryo-ET data including tomograms and segmentations are available on EMDB (EMD-56367) and EMPIAR (accession code EMPIAR-13326).

The experimental imaging data for the presented analysis, the used Spotiflow model with corresponding training data and example data set are available in the BioStudies database (accession number S-BIAD2611).

Raw numbers for plots presented in this paper as well as western blot images are available in the Source Data file. All other data and unique reagents that support this study are available from the corresponding authors upon request.

Research involving human participants, their data, or biological material

Policy information about studies with human participants or human data. See also policy information about sex, gender (identity/presentation), and sexual orientation and race, ethnicity and racism.

Reporting on sex and gender	n/a
Reporting on race, ethnicity, or other socially relevant groupings	n/a
Population characteristics	n/a
Recruitment	n/a
Ethics oversight	n/a

Note that full information on the approval of the study protocol must also be provided in the manuscript.

Field-specific reporting

Please select the one below that is the best fit for your research. If you are not sure, read the appropriate sections before making your selection.

☒ Life sciences ☐ Behavioural & social sciences ☐ Ecological, evolutionary & environmental sciences

For a reference copy of the document with all sections, see nature.com/documents/nr-reporting-summary-flat.pdf

Life sciences study design

All studies must disclose on these points even when the disclosure is negative.

Sample size	No statistical methods were used to determine necessary sample size, instead they were estimated based on recent studies using similar methodology.
Data exclusions	No data was excluded post analysis. Some analysis pipelines used internal quality checks to screen the raw input to assure the analysis of good quality data.

	For cryo-ET, tomograms of poor reconstruction quality or contrast (discontinuous or weak membrane and actin densities), typically originating from poor vitrification, lamellae regions thicker than 300 nm, or lamellae regions damaged during the milling, were not used for analyses. Actin and membrane segmentations were manually verified.
Replication	All biophysical and imaging experiments have been performed with at least 2 biological replicates, and almost all had at least 3 biological replicates. The sole exception is the cortical tension measurements performed on Cryo-ET grids, which were carried out once as a confirmatory validation of a consistently robust phenotype.<br>Cryo-electron tomography data were acquired from multiple, separate grids of cells grown and frozen on different days (a detailed list is provided in the manuscript).
Randomization	Where applicable, conditions were randomized by changing the order in which data was acquired to remove possible sources of bias connected to time. Cortical tension data collected in presence of the SMIFH2, IMM01, CK666 or Blebbistatin were always collected vehicle only first, followed by the treatment condition to avoid compound traces in the control condition.
Blinding	The actin segmentation was done blinded.

Reporting for specific materials, systems and methods

We require information from authors about some types of materials, experimental systems and methods used in many studies. Here, indicate whether each material, system or method listed is relevant to your study. If you are not sure if a list item applies to your research, read the appropriate section before selecting a response.

Materials & experimental systems

n/a	Involved in the study	
☐	☒	Antibodies
☐	☒	Eukaryotic cell lines
☒	☐	Palaeontology and archaeology
☒	☐	Animals and other organisms
☒	☐	Clinical data
☒	☐	Dual use research of concern
☒	☐	Plants

Methods

n/a	Involved in the study	
☒	☐	ChIP-seq
☐	☒	Flow cytometry
☒	☐	MRI-based neuroimaging

Antibodies

Antibodies used	anti-Phospho-MLC2 (Ser19), #3671, Cell signaling technology; anti-mDia1, #20624-1-AP, Thermo Fischer Scientific; anti-phospho-Ezrin (Thr567)/Radixin (Thr564)/Moesin(Thr558), #3141 Cell signaling technology; GAPDH, #NB300-221, Novus Biologicals; AF647 anti-rabbit #A21244, Thermo Fisher Scientific; Donkey-Anti-Rabbit-HRP, #711-035-152, Jackson Immuno Research; Goat-Anti-Mouse-HRP, #115-035-062, Jackson ImmunoResearch
Validation	All used antibodies were acquired commercially and validates by their producers.

Eukaryotic cell lines

Policy information about cell lines and Sex and Gender in Research

Cell line source(s)	NIH-3T3 and HL60 cells were purchased from ATCC; mouse embryonic stem cells provided by Austin Smith's group, Cambridge Stem Cell Institute
Authentication	n/a
Mycoplasma contamination	All cell lines were tested negative for Mycoplasma contamination
Commonly misidentified lines (See ICLAC register)	n/a

Plants

Seed stocks	n/a
Novel plant genotypes	n/a
Authentication	n/a

Flow Cytometry

Plots

Confirm that:

- [ ] The axis labels state the marker and fluorochrome used (e.g. CD4-FITC).
- [ ] The axis scales are clearly visible. Include numbers along axes only for bottom left plot of group (a 'group' is an analysis of identical markers).
- [ ] All plots are contour plots with outliers or pseudocolor plots.
- [x] A numerical value for number of cells or percentage (with statistics) is provided.

Methodology

Sample preparation	cell detachment; generation of single cell suspension in flow buffer (PBS supplemented with 0.5% BSA, 2.5mM EDTA) using a cell strainer
Instrument	LSRFortessa; FACSSymphony A5 (both BD BioSciences)
Software	FACSDiva software
Cell population abundance	Cell population abundance in reported experiment was high as the input sample originated from cell culture. Only debris and cell doublets were removed from the analysis via gating and sample purity evaluation was not required.
Gating strategy	A simple gating strategy was used as the sample originated from a pure culture. Debris and cell doublets were removed using FCS/SSC.

- [ ] Tick this box to confirm that a figure exemplifying the gating strategy is provided in the Supplementary Information.
